# Drawing Sensors with Ball-Milled Blends of Metal-Organic Frameworks and Graphite

**DOI:** 10.3390/s17102192

**Published:** 2017-09-23

**Authors:** Michael Ko, Aylin Aykanat, Merry K. Smith, Katherine A. Mirica

**Affiliations:** Department of Chemistry—Burke Laboratory, Dartmouth College, Hanover, NH 03755, USA; Michael.Ko.GR@dartmouth.edu (M.K.); Aylin.Aykanat.GR@dartmouth.edu (A.A.); Merry.K.Smith@dartmouth.edu (M.K.S.)

**Keywords:** gas sensor, chemiresistor, metal-organic framework

## Abstract

The synthetically tunable properties and intrinsic porosity of conductive metal-organic frameworks (MOFs) make them promising materials for transducing selective interactions with gaseous analytes in an electrically addressable platform. Consequently, conductive MOFs are valuable functional materials with high potential utility in chemical detection. The implementation of these materials, however, is limited by the available methods for device incorporation due to their poor solubility and moderate electrical conductivity. This manuscript describes a straightforward method for the integration of moderately conductive MOFs into chemiresistive sensors by mechanical abrasion. To improve electrical contacts, blends of MOFs with graphite were generated using a solvent-free ball-milling procedure. While most bulk powders of pure conductive MOFs were difficult to integrate into devices directly via mechanical abrasion, the compressed solid-state MOF/graphite blends were easily abraded onto the surface of paper substrates equipped with gold electrodes to generate functional sensors. This method was used to prepare an array of chemiresistors, from four conductive MOFs, capable of detecting and differentiating NH_3_, H_2_S and NO at parts-per-million concentrations.

## 1. Introduction

Portable electronic gas sensors are important for monitoring environmental hazards [1,2], ensuring home and workplace safety [3], increasing efficiency in food management [4,5], and protecting human health [6,7]. The demand for sensitive and selective detection of gaseous analytes has led to the development of diverse electronic device architectures including chemiresistors [8,9], chemicapacitors [10,11], field-effect transistors [10,12], and chemidiodes [13]. Compared to other architectures, chemiresistors are simple, low-cost, and low-power, while still capable of providing rapid analyses of surrounding gaseous environments [8,9]. Metal oxides [1,14,15], conductive polymers [16,17,18], carbon nanotubes [19,20], and metal nanoparticles [21,22] have all been shown to serve as effective materials in the chemiresistive detection of gases, but each class of these materials have unique limitations for achieving optimized selectivity and sensitivity towards target analytes. For instance, metal oxides typically operate at high temperatures [1,14,15], conductive polymers may rely on lengthy targeted monomer synthesis [3,8,16], and carbon nanotubes often require post-synthetic modifications to enhance their selectivity and sensitivity to target analytes [9,20]. Recently, research efforts to circumvent these challenges have focused on the development of new classes of chemiresistive materials capable of straightforward synthetic tunability. Materials prepared via self-assembly are particularly desirable, as selectivity for certain analytes may be enhanced through strategic selection of modular building blocks [23,24]. 

Metal-organic frameworks (MOFs) are crystalline materials constructed from molecular building blocks that may accommodate a wide range of physical and chemical properties required for chemical sensing [25,26,27]. Judicious selection of multi-dentate monomer ligands with well-defined geometries can impart tunable porosity, dimensionality, and high internal surface areas, leading to functional utility in gas sequestration and storage [25,26,27]. The selection of redox-active or highly conjugated ligands allows for the rational design of MOFs that are electrically conductive [28,29,30]. The added feature of conductivity, coupled with high surface area and modular design, greatly expands the potential functional utility of this class of MOFs in portable electronic chemical sensing [23,24,25], energy storage [31,32], electronics [33,34,35], and energy conversion [36,37,38]. In the context of portable electronic chemical sensing, MOFs that display conductivity offer an alternative strategy for the design of chemiresistive materials, with at least four distinct advantages. First, MOFs are synthetically accessible through “bottom-up” self-assembly using straightforward solution-phase techniques [39,40,41]. Second, the structure and properties of MOFs can be tailored through strategic selection of their monomeric subunits to achieve selective chemiresistive response towards analytes [42,43,44]. Third, the modular architecture of MOFs can enable rapid access to multifunctional properties within the same material (e.g., selective adsorption, conductivity, and chemiresistivity) through “bottom-up” molecular design [39,45,46,47]. Fourth, the porosity of MOFs can facilitate gaseous perfusion and facile transport of analytes through the material [48]. 

Conductive MOFs typically exhibit electrical conductivities ranging from 10^−13^ S/cm to 10^3^ S/cm [28]; these values are comparable in magnitude to conductive chemiresistive polymers (10^−12^ S/cm to 10^5^ S/cm) and functionalized carbon nanotubes [49,50,51]. This broad range of conductivities—which reflects the structural diversity and microcrystalline character of MOFs—poses a challenge for the development of general strategies for integrating conductive MOFs into electronic sensing devices. Although the capacity to transport charge is a an essential property of chemiresistors, effective chemiresistive performance is influenced by both the transduction mechanism [52,53] and device architecture [54,55], and is not determined by the conductivity of the material alone. The conductivity of the chemiresistive material, however, often determines the ease and efficiency with which it can be integrated into a solid-state device. To achieve effective device integration, two criteria must be met: (i) efficient contact of the chemiresistive material with the electrodes, and; (ii) reliable charge percolation pathway through the device. While highly conductive semiconductors can be easily integrated into simple device architectures [23,50], moderately and weakly conductive semiconductors can face a set of challenges with device integration. Ineffective contact of weakly or moderately conductive material to the electrodes and difficulty with maintaining an efficient conductive percolation pathway within the microscale or mesoscale device can compromise device performance by decreasing the signal-to-noise ratio, reducing the yield of functional devices during fabrication, and leading to false positive signals [20]. Thus, the development of effective and generalizable methods for incorporating MOFs into electronic devices is critical for producing portable sensors from these materials. 

Existing techniques for incorporating MOFs into chemiresistive devices include drop casting [23,56], direct assembly [24], and mechanical abrasion [56]. Solution-phase methods, however, require activation, careful solvent exchange, washing, or rigorous drying for consistent device performance [24,42,57,58]. Direct assembly may not be generally applicable on all surfaces because it requires favorable adhesive interactions between the MOF and the device substrate in the solvothermal reaction mixture, and has not yet been shown to be compatible with a broad range of devices architectures. Mechanical abrasion is an exceedingly straightforward method for nanomaterial device integration, but current examples are limited to one of the most conductive bulk MOFs (2 S/cm) [29], and this technique can be challenging to implement with less conductive materials. A method for material integration that takes advantage of the ease of mechanical abrasion without compromising device performance would be highly useful for integrating and rapidly prototyping MOFs in chemiresistors.

Herein, we address the challenges with integrating MOFs into electronic devices and describe a rapid and general method for the incorporation of conductive MOFs into functional chemiresistive gas sensors by mechanical abrasion. We utilize the condensation of metal salts with hexahydroxytriphenylene (HHTP) to produce a series of semiconductive isoreticular two-dimensional (2D) MOFs (M_3_HHTP_2_, M = Fe, Co, Ni, or Cu) that display chemiresistive properties. To achieve efficient contact of the sensing material with the electrodes within the device, we create a solid blend of MOF with graphite powder (M_3_HHTP_2_/graphite), and then integrate the M_3_HHTP_2_/graphite blend into devices by drawing. The role of graphite is threefold: (i) it produces a highly conductive blend that can facilitate the contact between the MOF and the metallic electrode in the device; (ii) it improves the electrical contact between the individual MOF crystallites, thus promoting an efficient charge percolation pathway within the device, and; (iii) it acts as a binder that facilitates smooth deposition of sensing material into a thin film by mechanical abrasion on the surface of the device. We demonstrate the utility of this method by fabricating an array of chemiresistive sensors using four semiconductive M_3_HHTP_2_/graphite blends (four probe conductivities in the form of a compressed pellet ranging from 3.8 × 10^−^^2^ to 9.8 × 10^−^^1^ S/cm). The resultant devices have the capability to detect and differentiate three gaseous analytes (NH_3_, NO and H_2_S) at ppm concentrations.

## 2. Materials and Methods

### 2.1. General Synthetic Procedure of Pure MOFs

**Figure 1:**
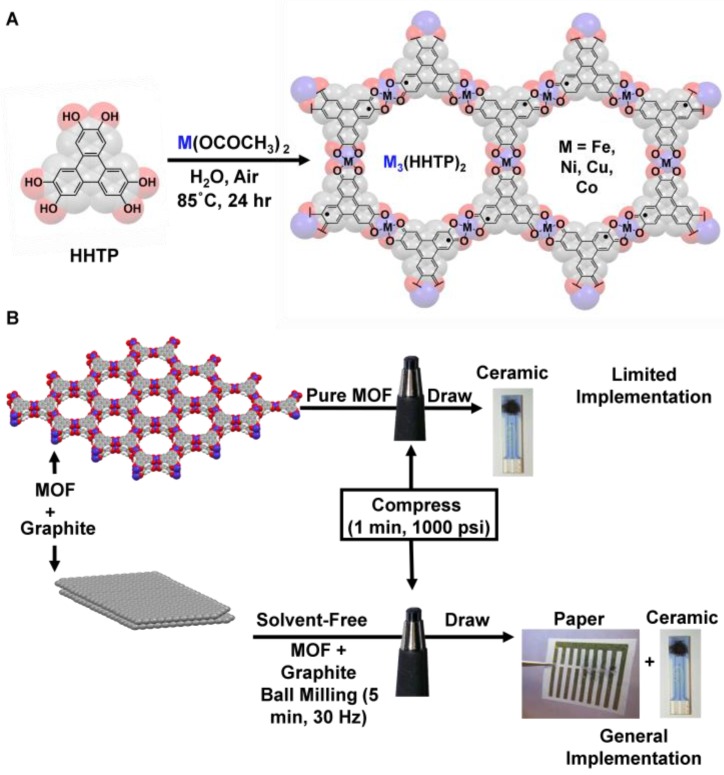
Synthesis of metal-organic frameworks (MOFs) and fabrication of sensors. (**A**) The synthetic scheme for the series of two-dimensional (2D) MOFs used in this study. (**B**) A schematic showing the stepwise process for integration of MOF-based materials into chemiresistive devices. Direct compression of the MOF and abrasion led to limited implementation in solid-state devices. Ball milling of M_3_HHTP_2_ MOF and graphite formed a blend that was subsequently compressed into a pellet. Loading of the pellet into a pencil-style holder, followed by mechanical abrasion directly onto paper or ceramic-based devices equipped with gold electrodes produced a series of chemiresistors with different architectures.

All chemicals and reagents were purchased from Sigma Aldrich (St. Louis, MO, USA) or TCI (Portland, OR, USA) and used without further purification. The synthesis of MOFs using organic linker 2,3,6,7,10,11-hexahydroxytriphenylene (HHTP) was adapted from Yaghi and coworkers (2012) (Figure 1A) [42]. In a 100 mL round bottom flask, the ligand HHTP (0.62 mmol) and metal acetate (1.23 mmol) were suspended in deionized water (28 mL). The mixture was sonicated (10 min) and then stirred at 85 °C (24 h). The flask was allowed to cool to ambient temperature over 2 h, and the precipitate was isolated by vacuum filtration. The solid product was washed with deionized water (3 × 50 mL) and with acetone (3 × 50 mL), transferred to a vial, and dried overnight under reduced pressure (4000 Pa) at 85 °C. 

### 2.2. Preparation of M_3_HHTP_2_/Graphite Blended Pellets

Ball milling of MOFs with graphite was conducted with a Retsch Mixer Mill, model MM400 (Retsch, Haan, Germany) at ambient conditions. The desired MOF (90 mg) and 99.995% pure graphite with average particle size of 2–15 μm (10 mg) (Alfa Asesar, Haverhill, MA, USA) were added to a 5-mL steel grinding jar with two steel balls (4 mm diameter). The mixture of both solids was milled at 30 Hz for 5 min. The pellets were prepared by adding the powdered M_3_HHTP_2_/graphite blends into a pellet die with a diameter of 6 mm (Across International, Livingston, NJ, USA) and applying a constant pressure of 6.9 MPa for 1 min using a Desktop Pellet Press (Across International, Livingston, NJ, USA). A 3-mm (custom made) cylinder was also compressed by applying a constant pressure 1.7 MPa for 1 min using a Desktop Pellet Press (Across International, Livingston, NJ, USA) at room temperature.

### 2.3. Methods of Sensing Device Preparation by Mechanical Abrasion

M_3_HHTP_2_/graphite blends were abraded into the gaps between the electrodes to generate chemiresistive devices. The 6-mm diameter pellet was abraded onto devices by holding between the index finger and thumb and physically pulled along the surface using an average force of 1–5 N. When connecting the gaps between the electrodes, we were careful to avoid deposition of the chemiresistive material elsewhere on the devices (Figure S1B). We also implemented a 3-mm diameter pellet placed into a pencil lead holder (Caran D’ache, Fixpencil 3 mm), and used this assembly to facilitate abrasion onto the surface of the substrate (Figure 1B and Figure S2C).

### 2.4. General Methods and Materials for Sensing Studies and Measurements of Chemiresistance

Chemiresistive devices were loaded into custom-made Teflon enclosures with five-pin caps to secure the devices (Figures S1D and S2D,E). These assemblies were connected to a mass flow controlled gas delivery system (Sierra’s Smart-Trak Series 100, Sierra Instruments, Monterey, CA, USA). Voltage was applied (0.1 V) to the devices and the current was monitored using a multichannel potentiostat (EmStat PalmSense EmStat-MUX-16, Palm Instruments BV, Houten, The Netherlands). Gaseous analytes were diluted in dry N_2_, and dosed through baseline (N_2_), exposure (diluted analyte, 5 min), and recovery (N_2_, 10 min) cycles. During the sensing experiments, all sensors were maintained at room temperature.

The mass-flow controllers were used to regulate the delivery of specific gas concentrations to the devices. Concentrations of 0.5–80 ppm were delivered using custom-mixed gas tanks (1% NH_3_, NO, H_2_S and CO diluted in N_2_, purchased from Airgas: Lebanon, New Hampshire) diluted with a flow of 500 mL/min of N_2_. For the purposes of determining upper limit of response, higher concentrations (up to 1250 ppm) of NH_3_ were delivered using pure NH_3_ diluted with a flow of 500–2000 mL/min of N_2_.

For vapors, the gas generator oven temperature was set to 40 °C to with a flow rate set to deliver 500 ppm vapor concentrations for MeOH, EtOH, and acetone. For H_2_O, the oven temperature was set to 80 °C with a flow rate set to deliver 7000 ppm vapor concentrations. A gas generator (KIN-TEK Flex Stream, La Marque, TX, USA) was used to deliver specific concentrations of vapors to the devices. Each vapor was calibrated, to determine specific concentrations, by measuring mass loss at a set flow rate and using the Equation below (1) [59].

PPM = [Weight loss (g) × 10^9^]/[total time (min) × molecular weight of vapor (g/mol) × flow rate (sccm)] (1)

### 2.5. Data Processing of Sensing Response

Conductance data collected was normalized using the following Equation (2) giving overall percent response. The current at the baseline is represented by “I_0_” and “I” represents the current at any point in time. 

−ΔG/G_0_ (%) = [(I_0_ − I)/I_0_] × 100% (2)

## 3. Results and Discussion

### 3.1. Characterization of Pure MOFs

We synthesized MOFs by reacting an organic ligand, 2,3,6,7,10,11-hexahydroxytriphenylene (HHTP), with metallic nodes (metal acetate, M = Fe, Co, Ni or Cu). The ligand coordinated to the metal in a 2:3 ratio, interconnecting the subunits and producing four semiconductive M_3_HHTP_2_ analogues with different metal centers (M = Fe, Co, Ni, or Cu) (Figure 1A and Figure S3). The resulting MOF powders were characterized using powder X-ray diffraction (pXRD), scanning electron microscopy (SEM), energy dispersive X-ray spectroscopy (EDS), thermal gravimetric analysis (TGA) and Brunauer-Emmett-Teller analysis (BET) (see supporting information). To test their conductivity, the MOF powders were compressed into pellets (~90 mg, diameter = 6 mm) using a hydraulic press (1000 psi, 1 min). Bulk conductivity (σ) of the pellets—quantified by a four-contact point linear probe—was found to be σ(Co_3_HHTP_2_) = 2.7 × 10^−^^6^ S/cm, σ(Ni_3_HHTP_2_) = 1.0 × 10^−^^1^ S/cm, σ(Cu_3_HHTP_2_) = 2.0 × 10^−^^2^ S/cm, and σ(Fe_3_HHTP_2_) = 3.0 × 10^−^^3^ S/cm (Table S1).

For Cu_3_HHTP_2_, Ni_3_HHTP_2_, and Co_3_HHTP_2_ MOFs, characterization matched reported analysis [24,42]. The pXRD analysis of these materials showed distinct peaks at 2θ = 4.5°, 9.4° and 12.4° corresponding to (100), (020) and (120) Miller indices, respectively (Figure S8). These peaks represent interatomic distances of 20, 9 and 7 Å, respectively, and correspond to long range pore:pore order within the crystal, as all three Miller indices run perpendicular to the 2D plane of the MOF. The plane (100) evenly bisects the pore such that the plane represents the interpore distance (19 Å as measured from .cif of M_3_HHTP_2_ in slipped parallel stacking generated from Materials Studio). Lattice plane (020) cuts through the molecular bulk along metal centers so that the plane matches the metal:metal distances (9 Å as measured from .cif of M_3_HHTP_2_ in slipped parallel stacking generated from Materials Studio). Finally, the plane (120) runs diagonally through alternating metal centers, triphenylene moieties, and pores (7 Å as measured from .cif of M_3_HHTP_2_ in slipped parallel stacking generated from Materials Studio). These pXRD results are in good agreement with the previously reported pXRD characterization of Cu_3_HHTP_2_, Ni_3_HHTP_2_ and Co_3_HHTP_2_ [24,42].

The BET surface area analysis using N_2_ gave values of 473 m^2^/g for Ni_3_HHTP_2_, 284 m^2^/g for Cu_3_HHTP_2_, and 571 m^2^/g for Co_3_HHTP_2_ (Figure S10). The surface area for Ni_3_HHTP_2_ MOF was similar to previously reported BET surface area measurement using Ar (425 m^2^/g) [42]. TGA indicated the decomposition of Ni_3_HHTP_2_ and Co_3_HHTP_2_ MOFs with mass loss between 57% and 79% at 750 °C, also matching reported characterization [42]. Thermal decomposition of Cu_3_HHTP_2_ showed a 31% mass loss, similar to reported results [24].

Characterization for Fe_3_HHTP_2_ coordination polymer, a novel material, included molecular simulation of possible crystalline configurations and comparison of the simulated pattern to experimental pXRD (Materials Studio). Experimental pXRD results suggested that Fe_3_HHTP_2_ was largely amorphous, with no distinct diffraction peaks compared (Figure S8). Analysis by SEM matched this observation, where globular, non-crystalline microstructures were evident. Elemental composition analysis by single-point EDS indicated the presence of the expected elements in correct distributions: the presence of iron was observed throughout the materials, suggesting a homogenous material (Figure S6). TGA showed a decomposition similar to the decomposition of the isoreticular MOF analogues (50% at 650 °C, Figure S9). The emergence of conductivity of the Fe_3_HHTP_2_ sample (Table S1) from its non-conductive monomers supported the formation of a new material. This cumulative evidence suggested that Fe_3_HHTP_2_ exhibited properties of a MOF-like coordination polymer, lacking sufficient crystallinity to be observed by pXRD. The BET analysis gave a surface area of 69 m^2^/g, consistent with the limited crystallinity and high amorphous character of the resulting material.

We initially attempted to integrate these pure MOFs directly into chemiresistive devices. Pellets of pure M_3_HHTP_2_ were prepared (diameter = 6 mm, Figure S4), and abraded directly onto the surface of commercial ceramic devices equipped with interdigitated gold electrodes (spacing = 180 μm). This technique was marginally effective in creating a continuous electrical pathway for charge transport within the devices. Subsequent attempts to use these devices as chemiresistive sensors were similarly challenging: the devices made with the pellets of Ni_3_HHTP_2_, Co_3_HHTP_2_ and Fe_3_HHTP_2_ consistently produced highly resistive pathways (3–30 MΩ) after abrasion with inefficient contact to the electrodes. Only one MOF used in this study—Cu_3_HHTP_2_—provided an adequate pathway for charge transport in the context of chemiresistive sensing. 

### 3.2. Characterization of M_3_HHTP_2_ MOF/Graphite Blends

With pure MOF pellets incapable of producing continuous conductive pathways via direct abrasion, we set out to improve the generality of the fabrication approach by using conductive additives. We created M_3_HHTP_2_/graphite blends (9:1 by mass) by ball milling the pure MOF with the graphite (30 Hz, 5 min), then compressed this blend into the form of a pencil (Figure 1B). This process was similar to that used by Mirica et al. in the preparation of carbon nanotube composites [50]. The pellets of M_3_HHTP_2_/graphite blends showed a significant increase in the bulk conductivity (3.8 × 10^−^^2^–9.8 × 10^−^^1^ S/cm) as compared to pure MOFs (2.7 × 10^−^^6^–2.0 × 10^−^^2^ S/cm) (Table S1). Pure MOFs had a range of bulk conductivities spanning four orders of magnitude due to the identity of the metal linkers, differences in crystal packing, and crystal size. In contrast, the bulk conductivity of the blends covered a much narrower range, falling within a factor of two. We hypothesized that blending of graphite with MOF powder before compression improved the electrical transport through the pellet by forming an efficient conductive junction between MOF crystallites. We expected the graphite to be minimally responsive towards chosen analytes with the MOF material dominating the sensing response, and graphite serving the function of a relatively inert conductive binder that promoted: (i) efficient contact between the sensing material and the metallic electrodes; (ii) continuous charge percolation pathway within the device, and; (iii) facile integration of the chemiresistive material into the device by mechanical abrasion.

**Figure 2:**
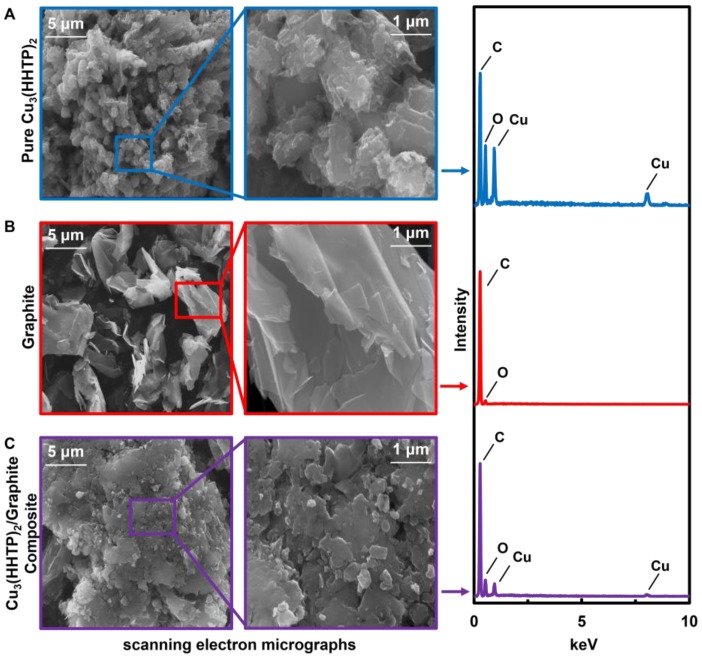
Scanning electron microscopy (SEM) micrographs and energy dispersive X-ray spectroscopy (EDS) analysis of pure MOFs, graphite, and Cu_3_HHTP_2_/graphite blend. From left to right: (**A**) Microcrystals of pure Cu_3_HHTP_2_ MOFs at 3200× and 20,000× magnification and corresponding EDS analysis; (**B**) Graphite at 3200× and 20,000× magnification and corresponding EDS analysis; (**C**) Cu_3_HHTP_2_/graphite blend at 5000× and 20,000× magnification and corresponding EDS analysis.

Characterization of the M_3_HHTP_2_/graphite blends by SEM and EDS mapping supported effective microscale mixing of MOF and graphite (Figure 2 and Figure S6, respectively). Using SEM, we determined that, before blending, the graphite existed as stacked sheets (6–12 μm in diameter or lateral dimensions), whereas the MOF crystallites appeared as aggregated clusters (2–3 μm in size). The blended material displayed significant differences in morphology when compared to the pure materials: in particular, there was a loss of uniformity with particle and sheet sizes ranging from 0.1 μm to 8 μm (Figure 2C), suggesting that the MOF coated the graphite crystallites (Figure S5). EDS mapping (Figure S6) supported this observation by indicating homogeneous distribution of metal and oxygen on the microscale. The TGA thermographs for Ni_3_HHTP_2_/graphite and Co_3_HHTP_2_/graphite blends exhibited a 3% mass loss at 100 °C, likely due to water, followed by a mass loss of 55% and 72% at 600 °C, respectively, very similar to the pure MOFs. In the case of Cu_3_HHTP_2_, both pure MOF and M_3_HHTP_2_/graphite blend demonstrated higher thermal stabilities than their isoreticular analogues [42] with only 31% mass loss at 600 °C. Minor differences in TGA were observed for Co_3_HHTP_2_ and Fe_3_HHTP_2_ between the pure MOF and the M_3_HHTP_2_/graphite blends as follows: 71% for Co_3_HHTP_2_, 65% for Co_3_HHTP_2_/graphite blend; 51% for Fe_3_HHTP_2_, and; 59% for Fe_3_HHTP_2_/graphite blend (Figure S9).

We observed important differences between blends and pure MOFs using pXRD measurements (Figure S8). The analysis of pXRD spectra showed attenuation of major crystalline MOF shear planes upon ball milling of MOFs with graphite, but a shear plane at 2θ = 26° (corresponding to (002) Miller index of graphite) was present for all the blends. This (002) plane represents an interatomic distance of 3.4 Å characteristic of the interplanar spacing between 2D sheet of graphite. This observation suggests that graphite crystallites remained intact and did not exfoliate within the blends upon ball milling. The plane at 2θ = 4.5° was lost after blending with graphite, except in the case of Cu_3_HHTP_2_/graphite. The attenuation of crystalline peaks corresponding to long range order, such as 2θ = 4.5° and 9.4° (corresponding to (100) and (020) Miller indices of MOF, respectively), suggested that blending caused a significant loss of crystallinity (Figure S8). Specifically, the long-range pore:pore order perpendicular to the 2D plane of the MOF was diminished.

The BET surface area measurements indicated that the blended materials retained some porosity. The BET surface areas in N_2_ for pure MOFs were measured to be as follows: 473 m^2^/g for Ni_3_HHTP_2_; 283 m^2^/g for Cu_3_HHTP_2_; 571 m^2^/g for Co_3_HHTP_2_, and; 69 m^2^/g for Fe_3_HHTP_2_ (Figure S10). Blending the MOFs with graphite lowered the surface area of the resulting blends in all cases: down to 337 m^2^/g for Ni_3_HHTP_2_/graphite (29% decrease); 65 m^2^/g for Co_3_HHTP_2_/graphite (89% decrease); 13 m^2^/g for Fe_3_HHTP_2_/graphite (81% decrease); 13 m^2^/g for Cu_3_HHTP_2_/graphite (95% decrease). This decrease in surface area can be attributed to both the loss of crystallinity (observed quantitatively by pXRD), and the partial blocking of pores by flakes of graphite. Importantly, the influence of mechanical blending on the reduction of surface area and crystallinity is dependent on the type of the material. For instance, Cu_3_HHTP_2_/graphite blend maintained some crystallinity, despite suffering a significant 95% reduction in surface area upon blending. In contrast, Ni_3_HHTP_2_/graphite blend showed a substantial loss of crystallinity, but maintained a relatively high surface area with 337 m^2^/g. While we can conclude that blending MOFs with graphite has the detrimental effects on both the crystallinity and surface area compared to their pure MOF analogs, these tradeoffs are offset by the increased conductivity of the blends, which can facilitate their integration into functional devices.

### 3.3. Fabrication of Sensing Devices

As historically demonstrated, the engineering of a functional abradable material takes ingenuity and multiple stages of development [60]. The abrasion-based deposition of a conductive material into a functional electronic device can be influenced by both the physical properties of the material and the surface of the substrate [61]. The parameters affecting the performance of such devices include the hardness of the compressed pellet [62], the morphology of the crystallites [63], the presence of grain boundaries after abrasion [64] and the relative work functions of the electrodes and the material [65]. Additionally, the surface roughness of the substrate and the spacings of the electrodes between which the material is deposited can influence the efficiency of the conductive percolation pathway and the final thickness of the sensing material [50,61]. During our initial approach to device fabrication, we observed Cu_3_HHTP_2_ to be the only MOF that could be reproducibly and efficiently abraded onto the surface of a ceramic device equipped with interdigitated gold electrodes without the addition of graphite. While the other MOFs occasionally produced a charge percolation pathway, this direct deposition approach was inconsistent in producing functional chemiresistors in our simple device architectures. Thus, we proceeded with the addition of graphite to generate chemiresistive sensors with facile, solvent-free integration of the sensing material. 

This study focused on the fabrication of two different chemiresistive device architectures equipped with chemically inert gold electrodes. The first set of prototypical devices was generated to test the applicability of mechanically abrading the M_3_HHTP_2_/graphite blends. For simplicity, we chose commercially ceramic devices (BVT Technologies, CC1 Sensor) (Figure 1B and Figure S1) with gold electrodes in an interdigitated pattern (~220 μm). Once the feasibility of the abrasion was established, we expanded the scope of the method to architectures featuring arrayed devices on paper (Figure S2A) featuring gold electrodes in a gap pattern (1 mm). Paper was chosen as the substrate for its simplicity and demonstrated efficacy as a chemiresistive sensor substrate [61].

We aimed to achieve a consistent resistance range of 300–600 kΩ within each device. In the prototype ceramic devices, each device was prepared individually. For the full arrayed paper devices, 15 devices on a single paper substrate constituted a single array, which we henceforth refer to as a “chip”. For these chips, gaps were filled in an array fashion, with all four isoreticular M_3_HHTP_2_/graphite (M = Fe, Co, Ni and Cu) blends in a single chip deposited in triplicate. On paper chips, the deposition of abraded blends produced an average film thickness of 0.4 μm (Table S2), and these devices exhibited ohmic behavior from the range from −2.0 V to +2.0 V (Figure S13).

### 3.4. Sensing Results

#### 3.4.1. Comparison of Chemiresistive Sensing Performance of Cu_3_HHTP_2_, Ball-Milled Cu_3_HHTP_2_, Cu_3_HHTP_2_/Graphite Blends

**Figure 3:**
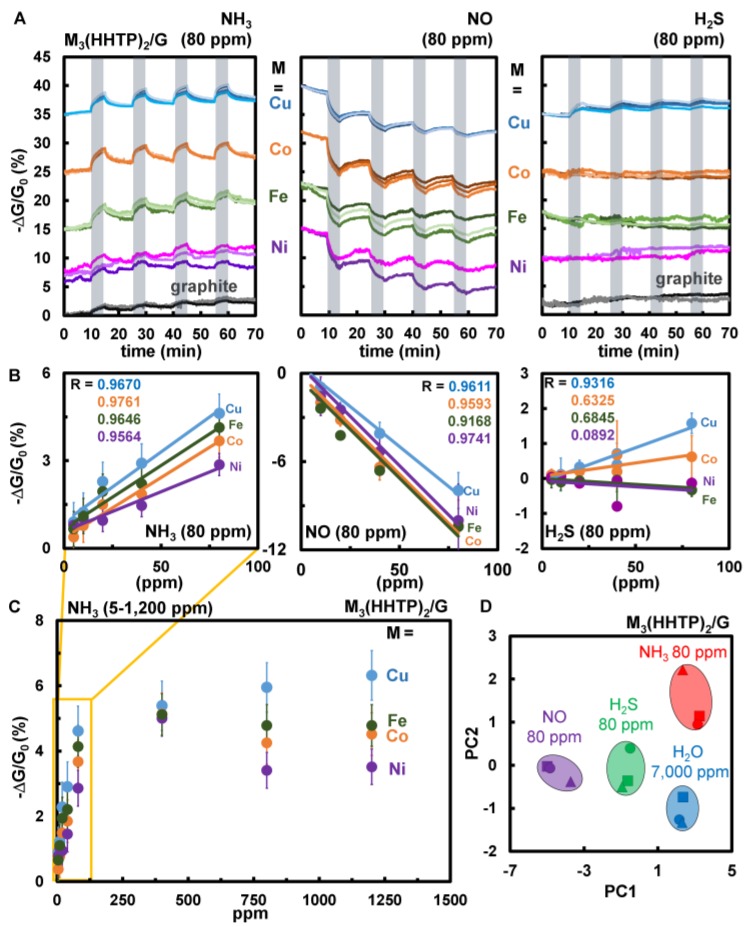
Sensing performance of M_3_HHTP_2_/graphite blends as chemiresistors when exposed to gaseous analytes, and statistical analysis of sensing response. (**A**) Representative sensing traces showing the change in conductance −ΔG/G_0_ (%) over time (min) for the M_3_HHTP_2_/graphite blends (Fe_3_HHTP_2_ = green, Co_3_HHTP_2_ = orange, Ni_3_HHTP_2_ = purple, Cu_3_HHTP_2_ = blue). The blends were abraded on paper devices between gold electrodes, then exposed to three different gases (80 ppm) diluted with N_2_ at room temperature. (**B**) Concentration dependence plots of sensing response of the M_3_HHTP_2_/graphite blends to NH_3_, NO and H_2_S (5–80 ppm). The error bars represent the standard deviation from the average. (**C**) Sensing performance of M_3_HHTP_2_/graphite array towards varying concentrations of NH_3_ (1200, 800, 80, 40, 20, 10, 5 ppm) diluted with N_2_. Each concentration was dosed for five minutes, followed by 10-min recovery. A linear increase in response to NH_3_ is observed through 80 ppm exposure, with a saturation limit occurring higher that 80 ppm. Subsequent exposures to NH_3_ at concentrations higher than 80 ppm show only a very small increase in response. (**D**) Principal component analysis of the data presented in Figure 3A demonstrates the ability of the array to distinguish between 80 ppm NH_3_, H_2_S, NO, and 7000 ppm H_2_O.

Our initial sensing studies used commercial ceramic device architectures to compare the chemiresistive performance between pure MOF, ball-milled MOF, and the M_3_HHTP_2_/graphite blend. Since only the Cu_3_HHTP_2_ showed a conductive pathway sufficient for sensing studies without additives in this architecture, sensing performance for pure Cu_3_HHTP_2_ MOF was compared to ball-milled Cu_3_HHTP_2_, and to the Cu_3_HHTP_2_/graphite blend. Three ceramic sensing devices were prepared for each sample, and all nine were dosed with MeOH (4 × 500 ppm). Thus, we proceeded to examine the generality of this approach across different M_3_HHTP_2_/graphite blends and different device architectures. The sequence of exposure was vapor followed by gas, but prior exposure of Cu_3_HHTP_2_/graphite devices to MeOH did not have a significant impact on their performance (Figure S20). The devices were preconditioned with MeOH for two reasons: (i) it is a frequently utilized organic solvent in an industry or laboratory setting, and; (ii) it was used as a model vapor to assess whether the sensing response of the device to NH_3_, NO and H_2_S gases would be altered after exposure to a polar, protic solvent such as MeOH. The shelf life stability of the blended materials was not explicitly studied, however the Cu_3_HHTP_2_/graphite blend was used to sense one analyte (NH_3_, H_2_S, NO, CO, Acetone, MeOH, EtOH and H_2_O) per day (Figure 3A and Figure S17). The devices were able to withstand eight days of sensing with different analytes without a decrease in device performance, showing short-term stability of the devices. Storage of the Cu_3_HHTP_2_/graphite blend in a vial under ambient conditions for six months decreased the conductivity of the material from 2.8 × 10^−1^ S/cm to 3.5 × 10^−2^ S/cm (Table S1), but the influence of this change on the sensing performance of the material was not studied.

#### 3.4.2. Chemiresistive Sensing Performance and Differentiation of NH_3_, NO, and H_2_S Using the M_3_HHTP_2_/Graphite Blends

To develop a general approach for integrating moderately conductive MOFs into chemiresistive devices, we prepared paper-based chips equipped with gold electrodes. We then mechanically abraded blended sensing materials onto paper-based chips for comprehensive sensing performance studies. Each chip featured all four blends (M_3_HHTP_2_/graphite, M = Fe, Co, Ni and Cu) in triplicate and pure graphite as a control. We discovered that an array comprising the four chemiresistors, detected and differentiated 80 ppm NH_3_, NO and H_2_S diluted in N_2_ (Figure 3 andFigure S15). We observed net trends in response of the array (averaged combined responses for M_3_HHTP_2_/graphite, M = Fe, Co, Ni and Cu) to each analyte. For NH_3_ (80 ppm), the resistance of the M_3_HHTP_2_/graphite array increased by an average of 2.1 ± 0.6%, while an opposite trend, a decrease in resistance, was observed when exposed to NO (80 ppm), with an average response of −1.8 ± 0.4%. The response of the array to H_2_S (80 ppm) is unique from NH_3_ and NO, with only one component—Cu_3_HHTP_2_/graphite—exhibiting a significant response with a decrease in resistance of 1.0 ± 0.5%. Principle component analysis [24,61] (Figure 3D) was employed to test the ability of the M_3_HHTP_2_/graphite array to distinguish four analytes (NH_3_, H_2_S, NO and H_2_O). The peak heights seen in Figure 3A were calculated by subtracting the highest point value from the baseline. These values were plugged into a Microsoft Excel function using the statistical analysis software called Analyse-it, which calculated principal component scores [62,66,67]. This statistical analysis correlated different components into groups, and formed statistically related clusters. The first two component scores accounted for 100% of the variance (Tables S5 and S6). This statistical analysis revealed that the array was capable of differentiating NH_3_, H_2_S, NO (80 ppm) from each other, and from water (7000 ppm).

#### 3.4.3. Limits of Detection and Dynamic Range

We then tested the effective sensing concentration range of the M_3_HHTP_2_/graphite array. The average responses of each separate M_3_HHTP_2_/graphite sample in the array exhibited a linear relationship to decreasing concentrations of analyte (5–80 ppm, Figure 3B). The M_3_HHTP_2_/graphite array not only exhibited analyte-dependent sensing responses, but also analyte-specific effective limits of detection (LOD): 17 ppm for NO; 19 ppm for NH_3_, and; 35 ppm for H_2_S. These LODs would be sufficient for detecting the Occupational Health and Safety Administration’s (OSHA) eight-hour permissible exposure limits (PEL) of NO and NH_3_ at 25 ppm [68]. While the LOD for H_2_S was found to be above the 10 ppm PEL for this gas over a corresponding eight-hour shift, it would be sufficient to detect the ceiling concentration of 50 ppm (over 10 min) [68].

We found that the sensor response of Cu_3_HHTP_2_/graphite blend approached full saturation at 100 ppm NH_3_ (Figure 3C). The overall sensitivity of the blended MOFs is lower when compared to other chemiresistive materials such as carbon nanotubes (3 ppb) [69] and carbon-based polymers (0.22 ppm) [70]. While the devices described herein cannot compete with this level of sensitivity, the dynamic range (5–1200 ppm) of MOF/graphite blends in detecting NH_3_ is comparable to that of metal oxide sensors [71], which is an impressive feature for devices that were fabricated with a rapid prototyping technique.

#### 3.4.4. Reproducibility Study and Scale Dependent Analysis of Cu_3_HHTP_2_/Graphite Blend

To investigate the effects of batch-to-batch reproducibility on device performance we compared two independently prepared batches of Cu_3_HHTP_2_/graphite blends. Both batches comprised Cu_3_HHTP_2_ MOFs prepared on a 200 mg scale (HHTP). In both experiments, the batches were integrated into paper devices (three devices per batch), exposed to analytes, and their chemiresistive performance compared. Device-to-device reproducibility within batches was in agreement: each device exhibited comparable sensing performance when exposed to NH_3_ (80 ppm) and MeOH (500 ppm, Figure S18). The average sensing response of Cu_3_HHTP_2_/graphite for batch 1 to NH_3_ was 2.3 ± 0.2%, while batch 2 produced an average response of 1.8 ± 0.5%. Statistical analysis with 95% CI showed the average responses, for batch 1, to fall between 2.1% to 2.4% and 1.3% and 2.4% for batch two. The overlap in the intervals suggests that the sensing responses between batch 1 and batch 2 have reproducible responses. When Cu_3_HHTP_2_/graphite was exposed to MeOH, the average response for batch 1 was 1.3 ± 0.1% with a 95% CI of 1.2% and 1.4%. For batch 2 the average sensing response to MeOH was 1.8 ± 0.4% with a 95% CI of 1.4% and 2.2%. For the two batches exposed to MeOH, the interval range is within overlap, which shows the devices are close to having reproducible response.

Scale-dependent changes in crystalline morphology were observed for Cu_3_HHTP_2_. The first batch comprised Cu_3_HHTP_2_ MOFs prepared on a 200-mg scale (HHTP), while the second was prepared at the 800 mg scale (HHTP). From each batch, 90 mg of Cu_3_HHTP_2_ was ball milled with 10 mg of graphite forming the blends. We observed a small difference in the sensing response of Cu_3_HHTP_2_/graphite blend to 80 ppm NH_3_ between batches of 2.5 ± 0.2% (for small-scale batch synthesized on 200 mg scale of HHTP) and 3.7 ± 0.6% (for large-scale batch synthesized on 800 mg scale of HHTP). These averages sensing responses had a 95% CI between 2.3 to 2.7% for batch 1 and 3.0% to 4.4% for batch 2, suggesting that sensor performance of Cu_3_HHTP_2_ may have some scale dependence. This difference in sensing response may be attributed to less consistent crystal size morphology in the large scale reaction compared to the small scale reaction, which was shown with SEM analysis, or to a difference in composition of the bulk isolate. Based on these results, a smaller scale synthesis was used for future experiments.

#### 3.4.5. Proposed Chemiresistive Sensing Mechanism 

**Figure 4:**
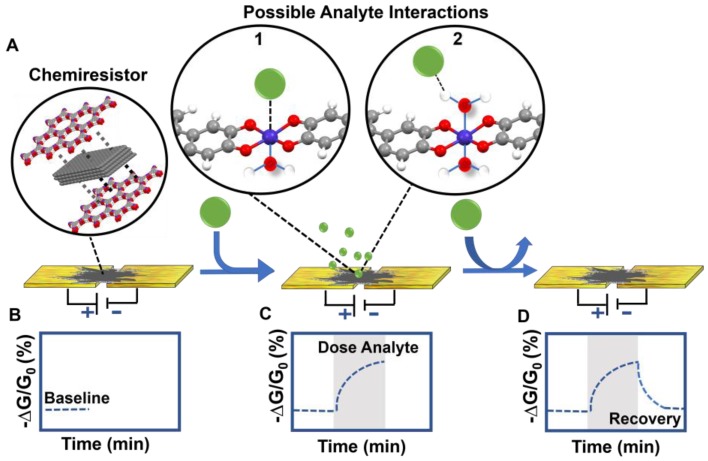
Schematic diagram of a MOF-based chemiresistor and possible analyte interactions. (**A**) A chemiresistive material (i.e., MOF/graphite blend) completes a circuit by bridging two electrodes. (**B**) The initial sensing trace (dashed blue line) shows a baseline (change in normalized conductance over time). (**C**) The chemiresistive device is exposed (grey shading indicates time of analyte dose) to an analyte (green circle) which can interact with the chemiresistive material by coordinating to the metal center (1), hydrogen bonding to the water at the axial position (2) or other binding modes. Perturbation to the charge transport through the nanomaterial leads to a change in conductance coincident with analyte binding. (**D**) In reversible binding modes, exposure to an inert carrier gas displaces the analyte, corresponding to the recovery of conductance back towards the original baseline.

Given the consistent sensing performance of materials, we posit that the distinct sensing responses produced by the chemiresistive materials reported herein are unique to the chemical interaction between each analyte and the materials within the array (Figure 4). The precise mechanism of how the analyte/sensor interaction impacts the charge transfer within the MOF is highly complex and not yet fully understood [56], but inferences can be made based on the single crystal data for Co_3_HHTP_2_, collected by Hmadeh et al. [42]. The coordination of the metal center in the network is octahedral, featuring equatorial coordination by the catecholates and two axial aqua ligands (Figure 4). Consequently, the options for the interactions with the analyte may include hydrogen bonding to the aqua ligands, coordinative displacement of the aqua ligands (Figure 4A), or charge transfer facilitated by the redox-active organic triphenylene-based building blocks within the MOF. These interactions can induce a change in the baseline conductance (Figure 4B), leading to a positive or negative change (Figure 4C). Further complexity is added by the potential presence of defects [42], the lack of information about the chemistry of the leading edges of the MOF crystallites, and the redox-active triphenylene ligands, which likely exist in the semiquinone state [72]. Furthermore, the mechanism of sensing is highly dependent on the reductive or oxidative capabilities of the analyte, which will lead to differences in sensing response in both direction and magnitude. Elucidating the sensing mechanism through consideration of these complex molecular-level interactions will require further spectroscopic investigation and cannot be made by observation of the chemiresistive sensing traces alone. Our results, however, along with those of others [23,24,29,56,73,74], provide a starting point for fundamental elucidation of host-guest activity in this class of materials. 

## 4. Conclusions

In conclusion, we developed a straightforward process for integrating conductive, triphenylene-based metal-organic frameworks (M_3_HHTP_2_) into chemiresistive sensors by mechanical abrasion of M_3_HHTP_2_/graphite blends directly into solid-state device architectures. The resulting sensors are capable of detecting and differentiating biologically and occupationally relevant toxic gases at ppm concentrations. The use of graphite as a blended facilitator greatly expands the scope of mechanical abrasion as a method for deposition in two ways: (i) by diversifying the range of nanomaterials compatible with the mechanical abrasion technique, and; (ii) promoting the deposition of materials on diverse substrates in different device architectures.

The simple strategy for device fabrication reported herein has enabled an expansion of chemiresistive chemical sensing into previously unavailable motifs. The value in this contribution is threefold. First, we envision that the technique of blending electrically accessible components with low-conductivity materials will be broadly applicable to a wide range of semiconductors, and potentially to insulators, as well. This strategy has the potential to expand the scope of porous materials in portable chemical sensing. Second, the blended pellets enable the direct deposition of the MOFs on the surface of electrodes by mechanical abrasion: a method that can be applied to diverse device substrates. Third, the integration of the MOF-based sensing material into devices is entirely solvent-free, which can preserve device substrates sensitive to harsh solvents.

The main limitation of the approach described herein is centered on the limits of detection of the analytes, which currently cannot compete with those of chemiresistors employing materials such as metal-oxides and conductive polymers [75,76,77]. The limited performance of the chemiresistive array could be linked to the amorphization and densification of the MOF and graphite ball-milled products, leading to blocked pores and reduced active surface area for analyte interaction. We envision that this limitation may be overcome by engineering highly conductive nanomaterials, or by optimizing device architectures for amplified responses [78]. Despite the high limits of detection, we believe that this fabrication method is highly valuable for rapid prototyping of MOFs as chemiresistors: the method can rapidly show the selectivity of low-conductivity MOFs to desired analytes at parts-per-million and part-per-thousand concentrations. Moreover, the rapid analysis of MOF selectivity for certain analytes leads to an intriguing possibility of utilizing MOFs as selectors for chemiresistive blends that employ a higher conductivity material as the transduction matrix (e.g., carbon nanomaterials). 

## Supplementary Materials

The following are available online at www.mdpi.com/1424-8220/17/10/2192/s1. Figure S1: Photographs showing the process of deposition of MOFs onto ceramic devices and integration into sensing setup. A) Ceramic device equipped with interdigitated gold electrodes. B) Mechanical abrasion using a 6 mm M3HHTP2/graphite blend pellet. C) Custom-made substrate holder for ceramic devices. D) Custom-made Teflon enclosure for sealed gaseous analyte exposure, Figure S2: Photographs showing the process of fabrication of paper devices and sensing setup. A) Weighing paper substrate with evaporated gold electrodes (1 mm gap). B) M3HHTP2/graphite pellet (6 mm) abraded onto paper-based chemiresistive device. C) M3HHTP2/graphite pellet (3 mm) abraded onto paper-based chemiresistive device using a mechanical pencil holder. D) Paper devices mounted onto a glass slide with double-sided tape. E) Paper devices on a glass slide inserted into Teflon enclosure. F) Teflon device enclosure clipped to 30 pin clip on a bread board, Figure S3: Scanning electron micrographs of Fe3HHTP2, Co3HHTP2, Ni3HHTP2, and Cu3HHTP2. Images of pure MOF crystallites, showing different morphology and size, Figure S4. Scanning electron micrographs comparing Cu3HHTP2 powder to compressed pellet form. SEM micrographs of pure MOF powder and compressed MOF pellet prepared by compression of powder at 1000 psi. Compression leads to increased contacts between the MOF crystallites, Figure S5: Scanning electron micrographs of Fe3HHTP2, Co3HHTP2, Ni3HHTP2, and Cu3HHTP2 graphite blends. A) Microcrystals of Co3HHTP2/graphite blend at 5,000x and 20,000x magnification. B) Fe3HHTP2/graphite blend at 5,000x and 20,000x magnification C) Ni3HHTP2/graphite blend at 5,000x and 20,000x magnification. D) Cu3HHTP2/graphite blend at 5,000x and 20,000x magnification, Figure S6: Energy dispersion spectrum mapping of Cu3HHTP2, graphite, and Cu3HHTP2/G blend. EDS mapping of Cu3HHTP2/graphite blend, Cu3HHTP2, and graphite. Each column shows an SEM image along with the corresponding EDS image to visually map characteristic X-rays for copper, carbon, and oxygen, Figure S7: Energy dispersive X-Ray spectroscopy of MOFs. Energy dispersive X-ray spectra of M3HHTP2 and M3HHTP2/graphite Blends. A) Fe3HHTP2, B) Co3HHTP2, C) Ni3HHTP2, and D) Cu3HHTP2, Figure S8: Powder X-Ray diffraction. Scaled powder X-Ray Diffraction (pXRD) spectra for graphite, M3HHTP2, and M3HHTP2/graphite blend bulk. The graphite peak at ~26° represents the interplanar (002) shear plane, corresponding to the stacking of 2D graphitic sheets. This peak is retained in each M3HHTP2/graphite blend, implying that graphite interplanar layers are not fully exfoliated upon ball-milling. Long-range crystallinity is diminished for the blends with the exception of Cu3HHTP2/G, which retains crystallinity after ball-milling with graphite. For Ni3HHTP2/G and Co3HHTP2/G, shear planes (100), (020), and (120)—all corresponding to Bragg planes perpendicular to the interplanar MOF layers—are attenuated upon ball-milling, suggesting significant loss of crystallinity upon milling. Fe3HHTP2 is amorphous in character before milling, Figure S9: Thermal gravimetric analysis (TGA). TGA curves for M3HHTP2 are represented by a solid line and M3HHTP2/graphite blends are represented by a dotted line. A) Fe3HHTP2, B) Co3HHTP2, C) Ni3HHTP2, and D) Cu3HHTP2. A 2–3 % mass loss is observed at 100° C, Figure S10: N2 isotherm. A) The isotherm plot for Ni3HHTP2 is shown in purple, Cu3HHTP2 in blue, Co3HHTP2in orange and Fe3HHTP2 in green. The solid circle represents the adsorption plot whereas the open circle corresponds to the desorption plot. The significant uptake under 0.1 (P/Po) is characteristic of a microporous material. The Brunauer-Emmet-Teller (BET) surface area for Ni3HHTP2 was calculated to be 473 m2/g. BET surface area for Cu3HHTP2 was calculated to be 284 m2/g. BET surface area for Co3HHTP2 was calculated to be 570 m2/g. BET surface area for Fe3HHTP2 was calculated to be 69 m2/g. The fitting range for BET calculations were 0 to 0.3 P/Po. B) The BET adsorption analysis for Ni3HHTP2/graphite (purple), Cu3HHTP2/graphite (blue), Co3HHTP2/graphite (orange) and Fe3HHTP2/graphite (green). The BET surface area for each of the blends was 337 m2/g, 13 m2/g, 65 m2/g, and 13 m2/g, respectively, Figure S11: A) The t-plot analysis (not fitted) using Harkins and Jura thickness equation for Ni3HHTP2 (purple), Cu3HHTP2 (blue), Co3HHTP2 (orange) and Fe3HHTP2 (green). B) The fitted t-plot analysis using the same thickness equation to calculate the external surface area. The external surface area for Ni3HHTP2 was calculated to be 79 m2/g, 55 m2/g for Cu3HHTP2, 158 m2/g for Co3HHTP2 and 65 m2/g for Fe3HHTP2. The blends exhibited decreased external surface areas of 118 m2/g for Ni3HHTP2, 8.1 m2/g for Cu3HHTP2, 33 m2/g for Co3HHTP2 and 11 m2/g for Fe3HHTP2, Figure S12: Enchantment of optical images to improve contrast of device image. Image taken with optical microscope and processed using ImageJ analysis to enhance contrast and calculate percentage of blue pixels for thickness, Figure S13: Current/voltage plots. Current/voltage plots demonstrate the ohmic behavior of the devices in the range of -2.0 V to 2.0 V. A) Cu3HHTP2/graphite blend device. B) Co3HHTP2/graphite blend device. C) Ni3HHTP2/graphite blend device. D) Fe3HHTP2/graphite blend device, Figure S14: Plot comparing sensing performance of pure MOFs with ball milled blends integrated by abrasion into ceramic devices equipped with gold-interdigitated electrodes. A) Sensing trace representing the change in conductance −ΔG/G0 (%) over time (min) with pure Cu3HHTP2, ball-milled Cu3HHTP2, and Cu3HHTP2/graphite blend exposed to MeOH (500 ppm) diluted with N2, using ceramic devices. B) Average sensing response of the three variants of copper MOF. Each bar represents the average value of response based on 4 exposures of 3 separate devices; the error bars represent the standard deviation from the average based on 4 exposures of 3 separate devices, Figure S15: Continuous concentration dependence analysis on M3HHTP2/graphite blends with varying concentration of NH3, NO, and H2S (80-5ppm). A) Sensing performance of M3HHTP2/graphite blend array towards varying concentrations of NH3, NO, and H2S (80, 40, 20, 10, 5 ppm) diluted with N2, exposed for five-minutes and 10-minute recovery times. A longer baseline is seen between the second and third exposures to allow for a proper baseline formation during a change from 0.5 L/min to 1.0 L/min. B) Plot of sensing response of the M3HHTP2/ graphite blends with respect to NH3, NO, and H2S at 5-80ppm. Each dot represents the average value of response based on 4 exposures of 3 separate devices; the error bars represent the standard deviation from the average, Figure S16: Saturation analysis on M3HHTP2/graphite blends with varying concentration of NH3 (5-8,000 ppm). Sensing performance of M3HHTP2/graphite blend array towards varying concentrations of NH3 (2,000, 1,600, 1,200, 800, 80, 40, 20, 10, 5 ppm) diluted with N2, exposed for five-minutes and 10-minute recovery times. A linear increase in response is observed through 80 ppm NH3 exposure with a saturation limit occurring after 80 ppm NH3 exposure. Subsequent exposures after 80 ppm NH3 only show a very small increase in response with higher doses of NH3, Figure S17: Plots showing response of arrays to different gases and vapors. Sensing performance of chemiresistive device array towards gaseous analytes. Sensing trace representing the change in conductance −ΔG/G0 (%) over time (min) with the M3HHTP2/graphite blends exposed to CO (80 ppm), EtOH, MeOH, and acetone (500 ppm), and H2O (7000 ppm) diluted with N2, Figure S18: Plot showing batch-to-batch reproducibility of chemiresistive sensors of M3HHTP2/graphite blends abraded between gold electrodes on paper. Sensing trace representing the change in conductance −ΔG/G0 (%) over time (min) with Cu3HHTP2/graphite blend abraded between gold electrodes on paper devices followed by subsequent exposure to NH3 (80 ppm) and MeOH (500 ppm). Blue represents the first batch (4 exposures with 3 devices) and red represents second batch (4 exposures with 3 devices). Average sensing response of Cu3HHTP2/graphite blend is plotted onto a bar graph with each bar representing the average percent response changed based on 4 exposures of 3 devices. The error bars represent the standard deviation from the average. A) Exposure to NH3 (80 ppm). B) Exposure to MeOH (500 ppm), Figure S19: Scale dependence of Cu3HHTP2 MOF morphology and sensing response. A) Small scale (200 mg of HHTP) Cu3HHTP2 MOF reaction shows an SEM image with nanorod morphology. Sensing trace shows a decrease in conductance with an average of 2.5% ± 0.2% change. B) Large scale (800 mg of HHTP) Cu3HHTP2 MOF reaction shows an SEM image with flake and small chunk morphology. Sensing trace shows a decrease in conductance with an average of 3.7% ± 0.6% change, Figure S20: Average response plot of Cu3HHTP2 MOF with no preconditioning versus preconditioning. Cu3HHTP2/graphite blend devices exposed to NH3 before or after MeOH exposure has no substantial difference. No precondition has blend devices exposed to NH3 first. Preconditioned blend devices are exposed to MeOH in a standard exposure trial (four exposures of 10 minutes with three recovery periods of five-minutes), Table S1: 4-point probe measurements. MOF pellets, 6 mm in diameter, measured for bulk conductance(S/cm) using a 4-point linear probe, Table S2: Table of BET surface areas for M3HHTP2 and M3HHTP2/Graphite, Table S3: Table of external surface areas for M3HHTP2 and M3HHTP2/Graphite, Table S4: Film Thickness of Materials on Devices. Table of areas, mass and density used in calculation for thickness of abraded layers, Table S5: Average sensory response for three arrays, excluding first exposures and graphite, Table S6: Principle Component scores for the three arrays featured in Table S4, high concentration of analyte, Table S7: Average sensory response for Cu3HHTP2/Graphite, excluding first exposures and graphite, Table S8: Average sensory response for Ni3HHTP2/Graphite, excluding first exposures and graphite, Table S9: Average sensory response for Co3HHTP2/Graphite, excluding first exposures and graphite, Table S10: Average sensory response for Fe3HHTP2/Graphite, excluding first exposures and graphite, Table S11. Signal-to-noise ratios of Cu3HHTP2/graphite, Cu3HHTP2 Ball-milled, and Cu3HHTP2.
