# Supplementary material for: Drawing Sensors with Ball-Milled Blends of Metal-Organic Frameworks and Graphite
Source: Sensors (Basel). 2017 Sep 23;17(10):2192. doi: 10.3390/s17102192 (PMC5677178; doi:10.3390/s17102192)
Supplement: Supplementary file 1 [file sensors-17-02192-s001.pdf]

Drawing Sensors with Ball-Milled Blends of Metal-Organic Frameworks and Graphite

Michael Ko, Aylin Aykanat, Merry K. Smith, Katherine A. Mirica*
Department of Chemistry – Burke Laboratory, Dartmouth College, Hanover, NH 03755, United States
*to whom correspondence should be addressed: katherine.a.mirica@dartmouth.edu

Supporting Information

Table of Contents S1

I. General Methods S2
II. Preparation of Chemiresistive Gas Sensors S3
III. Scanning Electron Microscopy of MOFs S5
IV. Four-Point Probe Measurements S8
V. Mapping of Materials with Energy Dispersive Spectroscopy (EDS) S9
VI. Energy Dispersive X-Ray Spectroscopy of MOFs S10
VII. Powder X-Ray Diffraction of MOFs S11
VIII. Thermal Gravimetric Analysis of MOFs S12
IX. Nitrogen Adsorption Measurements S13
X. Estimation of Thickness of the Abrasion Layer S16
XI. Current/Voltage Plots S18
XII. Comparison in Sensing Performance of Pure MOF with Ball Milled MOF/Graphite Blends S19
XIII. Analysis of Concentration Dependence S20
XIV. Saturation Response of Sensor Array with NH_3 S21
XV. Response of Sensor Arrays Comprising of MOF/Graphite Blends to Additional Gases and Vapors S22
XVI. Batch-to-Batch Influence of MOF/Graphite Blend for Chemiresistive Sensing S23
XVII. Scale-Dependent Cu_3HHTP_2 MOF Morphology and Sensing Response S24
XVIII. Influence of Previous Analyte Exposure on Subsequent Sensing Performance S25
XIX. Principle Component Analysis S26
XX. Variance Device/Device and Batch/Batch S27
XXI. Signal-to-Noise Analysis on Chemiresistive Response of Cu_3HHTP_2 and Cu_3HHTP_2/Graphite S29
XXII. References S30

I. General Methods

Weigh paper (Cat. No. 12578-121) was purchased from VWR International (Randor, PA). NH_3, NO and H_2S (1% concentration diluted in N_2) gas were purchased from Airgas (Radnor, PA). Commercial ceramic devices were purchased from BVT Technology (Brno, Czech Republic). Scanning Electron Microscopy (SEM) and Energy Dispersive X-ray Spectroscopy (EDS) was performed using a Hitachi TM3000 SEM (Tokyo, Japan) equipped for X-ray microanalysis with a Bruker Edax light element Si(Li) detector (Billerica, MA). Thermal Gravimetric Analysis (TGA) traces using a TA instruments TGA Q50 with platinum pans. Powder X-ray diffraction (pXRD) measurements were performed with a Bruker D8 diffractometer equipped with a Ge-monochromated 2.2kW (40kV, 40kA) CuKα (λ = 1.54 Å) radiation source and an NaI scintillation counter detector (Billerica, MA). Nitrogen adsorption measurements were performed with a ASAP 2020 Plus (Norcross, GA).

II. Preparation of Chemiresistive Gas Sensors

A. Chemiresistors on Ceramic Substrates

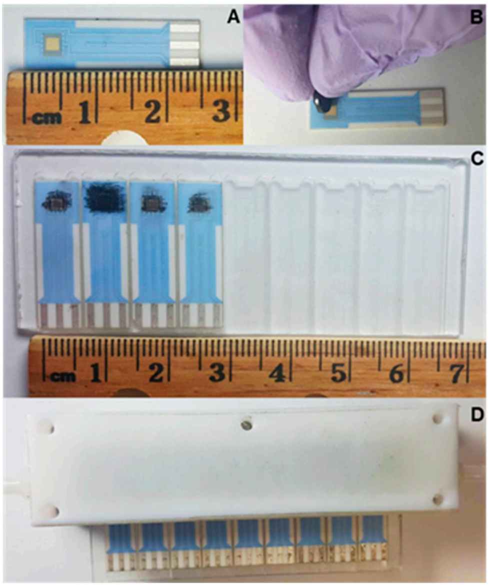

Figure S1. Photographs showing the process of deposition of MOFs onto ceramic devices and integration into sensing setup. A) Ceramic device equipped with interdigitated gold electrodes. B) Mechanical abrasion using a 6 mm M_3HHTP_2/graphite blend pellet. C) Custom-made substrate holder for ceramic devices. D) Custom-made Teflon enclosure for sealed gaseous analyte exposure.

B. Chemiresistors on Paper Substrates

Gold (99.995% purity) was deposited onto weighing paper (120 nm thickness) through a metal stencil mask with a 1 mm gap pattern (Angstrom Engineering, Ontario, Canada) using a Thermal Evaporator (Angstrom Engineering, Ontario, Canada) under a pressure of 0.5×10^{-5} Torr and a rate of evaporation of 1 Å/s.

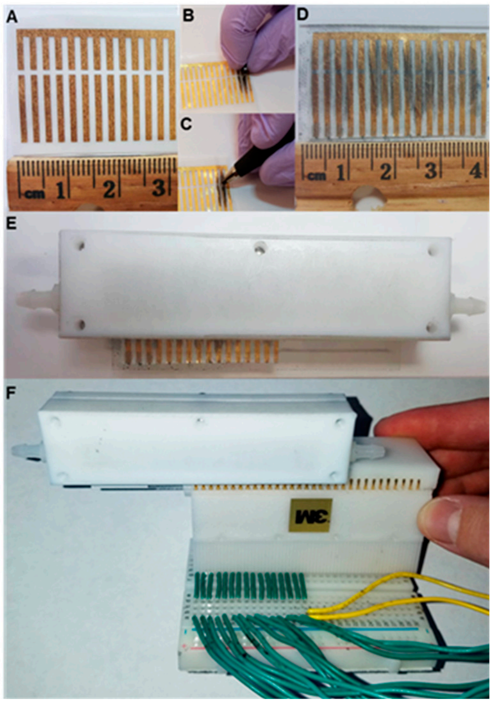

Figure S2. Photographs showing the process of fabrication of paper devices and sensing setup. A) Weighing paper substrate with evaporated gold electrodes (1 mm gap). B) M_3HHTP_2/graphite pellet (6 mm) abraded onto paper-based chemiresistive device. C) M_3HHTP_2/graphite pellet (3 mm) abraded onto paper-based chemiresistive device using a mechanical pencil holder. D) Paper devices mounted onto a glass slide with double-sided tape. E) Paper devices on a glass slide inserted into Teflon enclosure. F) Teflon device enclosure clipped to 30 pin clip on a bread board.

III. Scanning Electron Microscopy of MOFs

Scanning electron microscopy of bulk MOFs was obtained using a using a Hitachi TM3000 SEM with a 15.0 kV beam and working distance of 10 mm.

A. Pure M_3HHTP_2

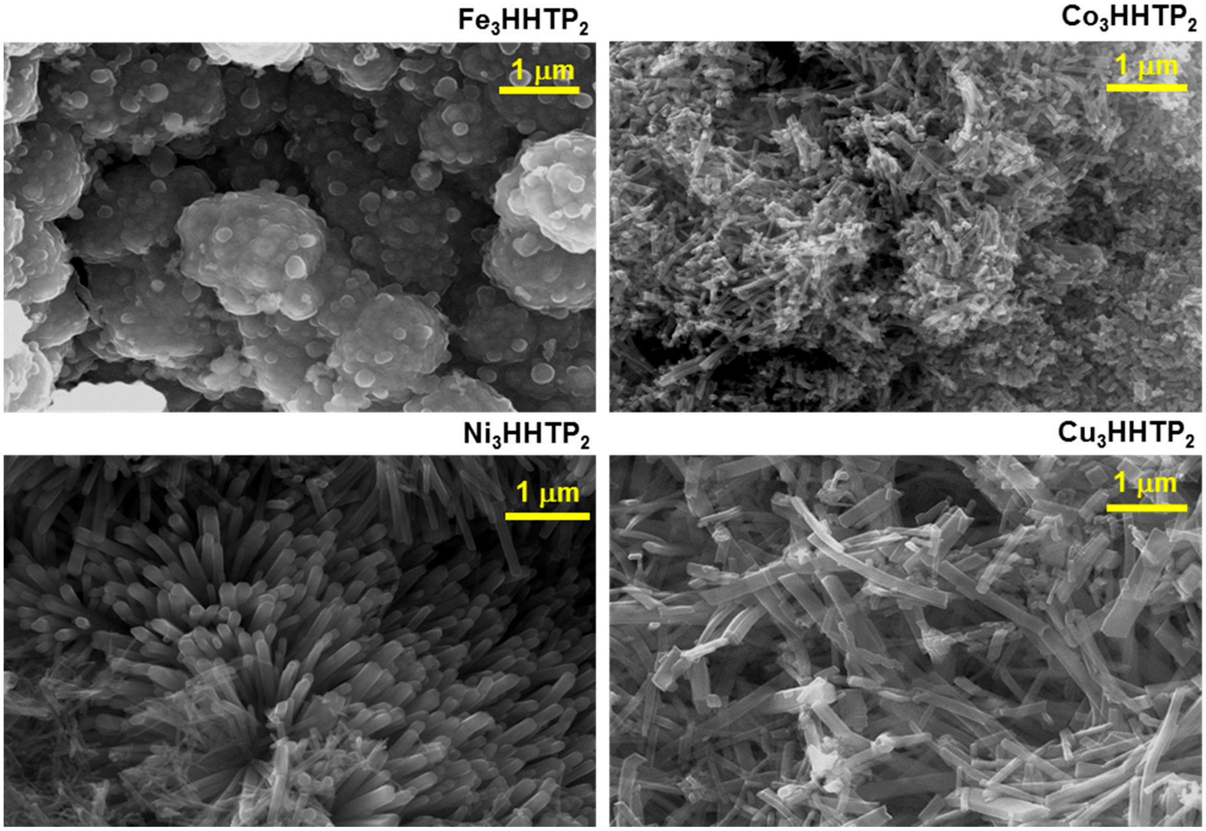

Figure S3. Scanning electron micrographs of Fe_3HHTP_2, Co_3HHTP_2, Ni_3HHTP_2, and Cu_3HHTP_2. Images of pure MOF crystallites, showing different morphology and size.

B. Comparison of Solid State Morphologies between Loose Powder and Compressed Powder Forms of Cu_3HHTP_2

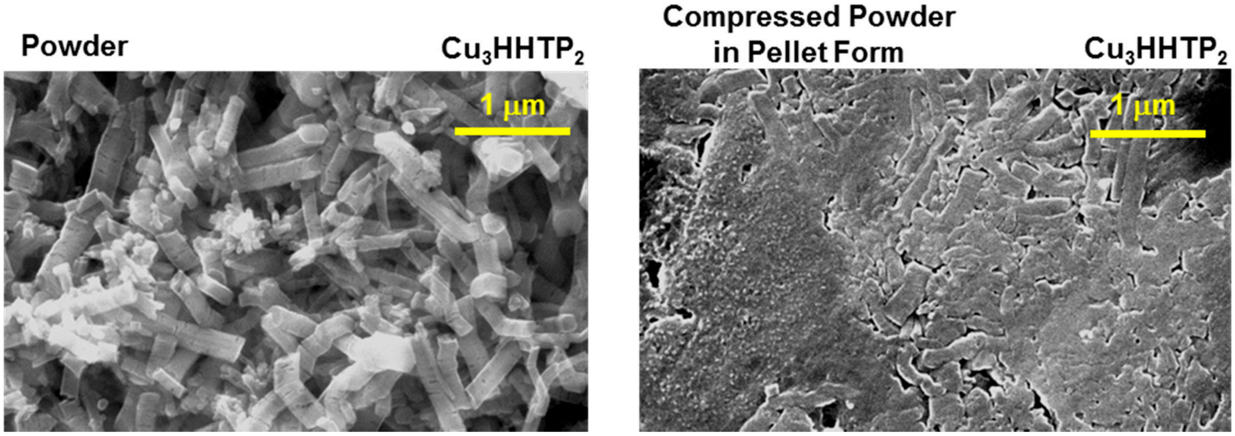

Figure S4. Scanning electron micrographs comparing Cu_3HHTP_2 powder to compressed pellet form. SEM micrographs of pure MOF powder and compressed MOF pellet prepared by compression of powder at 1000 psi. Compression leads to increased contacts between the MOF crystallites.

C. M_3HHTP_2/Graphite Blends

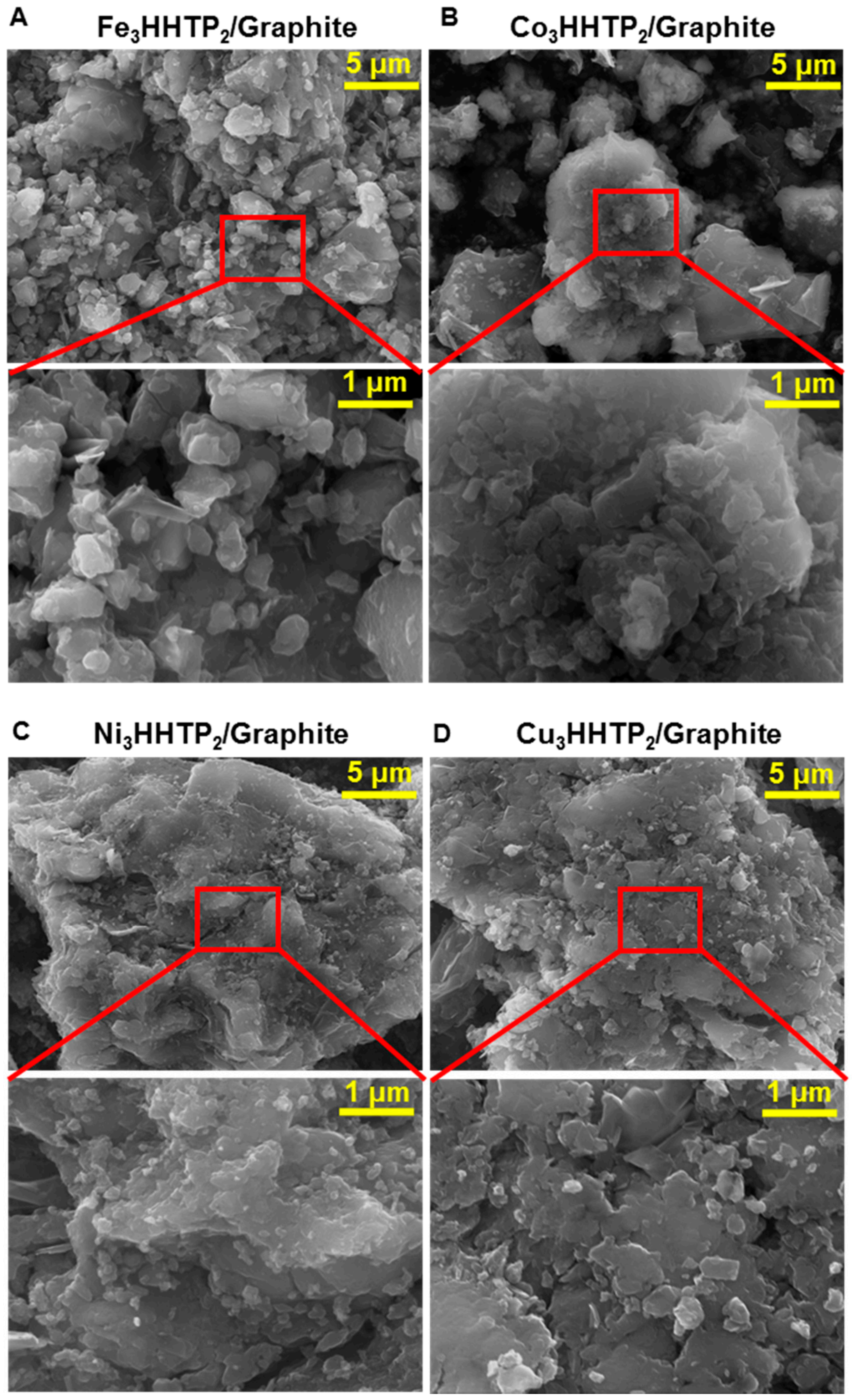

Figure S5. Scanning electron micrographs of Fe_3HHTP_2, Co_3HHTP_2, Ni_3HHTP_2, and Cu_3HHTP_2 graphite blends. A) Microcrystals of Co_3HHTP_2/graphite blend at 5,000x and 20,000x magnification. B) Fe_3HHTP_2/graphite blend at 5,000x and 20,000x magnification C) Ni_3HHTP_2/graphite blend at 5,000x and 20,000x magnification. D) Cu_3HHTP_2/graphite blend at 5,000x and 20,000x magnification.

IV. 4-Point Probe Measurements

A Singatone tungsten carbide four-point linear probe was employed to collect bulk conductance measurements of both pure MOFs and M_3HHTP_2/graphite blends with a space between tips of 1.27 mm. We calculated the bulk conductance measurements (S/cm) using equation (S1). The variables in the equations are I (A) is current, w (cm) is thickness of the pellet, C (unit less) is the correction factor accounting for the diameter of the pellet, and F (unit less) is the thickness correction factor that accounts for the thickness of a pellet.

$$\sigma = I/(V \times w \times C \times F) \tag{S1}$$

Table S1. 4-point probe measurements. MOF pellets, 6 mm in diameter, measured for bulk conductance(S/cm) using a 4-point linear probe.

	**Pure MOFs**	**M_3HHTP_2/G Blends**
**Fe_3HHTP_2**	3.0×10^{-3} S/cm	3.2×10^{-2} S/cm
**Co_3(HHTP$_2$**	2.7×10^{-6} S/cm	9.8×10^{-1} S/cm
**Ni_3HHTP_2**	1.0×10^{-1} S/cm	3.8×10^{-2} S/cm
**Cu_3HHTP_2**	2.0×10^{-2} S/cm	2.8×10^{-1} S/cm
**Cu_3HHTP_2**	-	3.5×10^{-2} S/cm (6 months shelf-life test)

V. Mapping of Materials with Energy Dispersive Spectroscopy (EDS)

EDS mapping of Cu_3HHTP_2/graphite blend were performed using SDD X-Ray microanalysis system with Octane Pro 10 sq. mm detector and TEAM software.

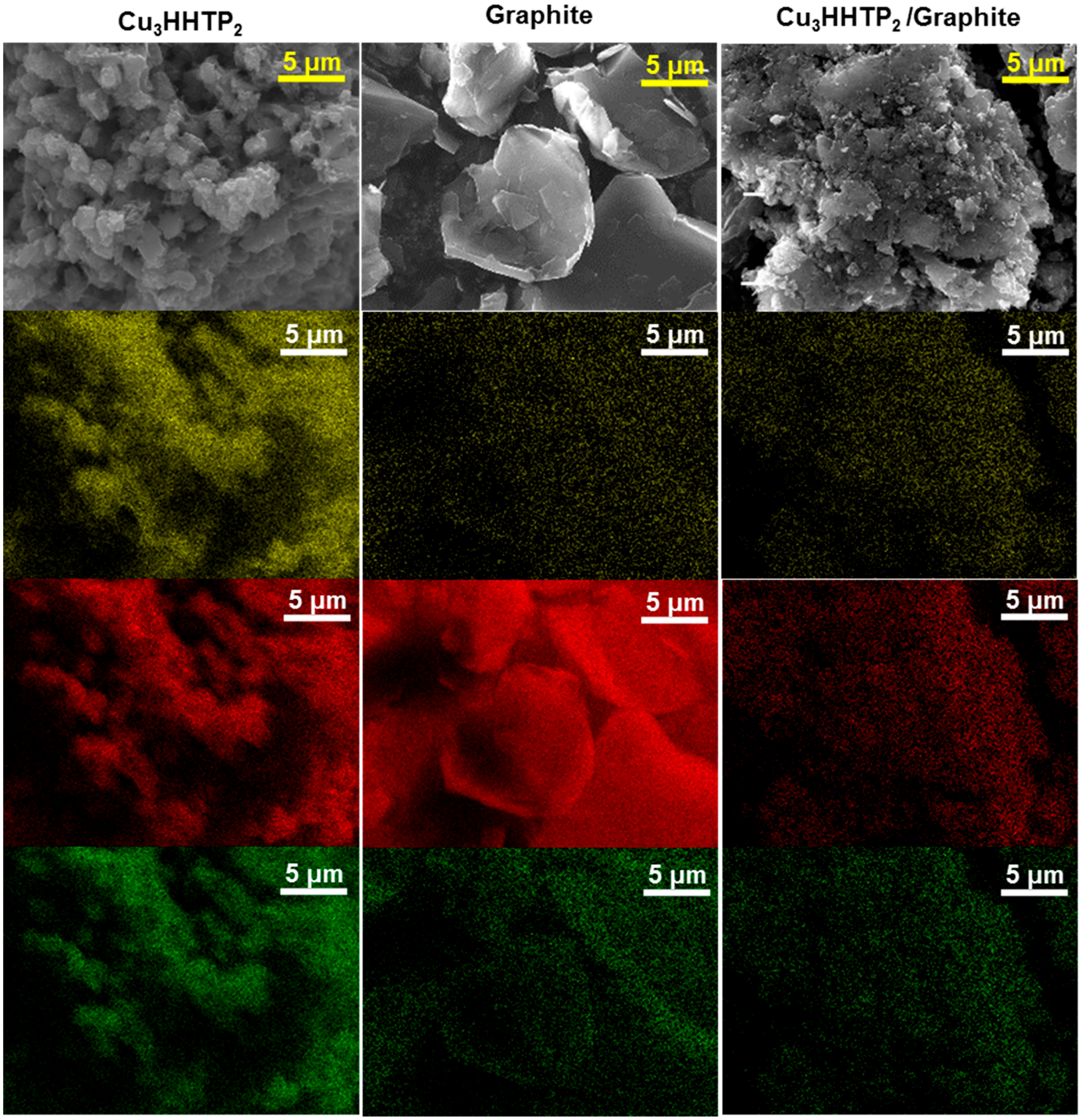

Figure S6. Energy dispersion spectrum mapping of Cu_3HHTP_2, graphite, and Cu_3HHTP_2/G blend. EDS mapping of Cu_3HHTP_2/graphite blend, Cu_3HHTP_2, and graphite. Each column shows an SEM image along with the corresponding EDS image to visually map characteristic X-rays for copper, carbon, and oxygen.

VI. Energy Dispersive X-Ray Spectroscopy of MOFs

Energy dispersive X-Ray spectroscopy was collected using SDD X-Ray microanalysis system with Octane Pro 10 sq. mm detector and TEAM software.

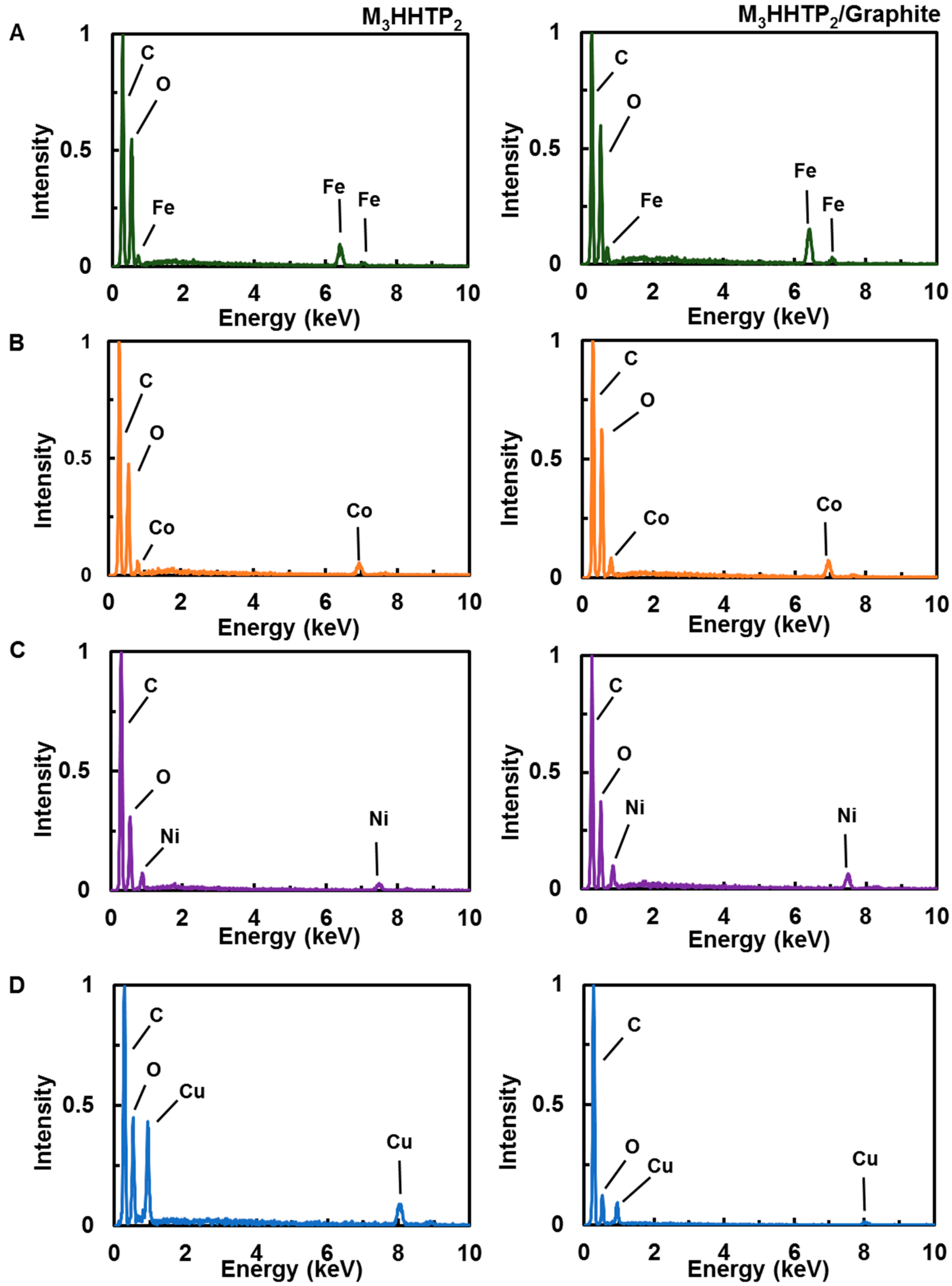

Figure S7. Energy dispersive X-Ray spectroscopy of MOFs. Energy dispersive X-ray spectra of M_3HHTP_2 and M_3HHTP_2/graphite Blends. A) Fe_3HHTP_2, B) Co_3HHTP_2, C) Ni_3HHTP_2, and D) Cu_3HHTP_2.

VII. Powder X-Ray Diffraction of MOFs

We collected spectra using a Bruker D8 Advance Powder X-ray Diffractometer (pXRD) equipped with a Ge-monochromated 2.2 kW (40kV, 40kA) CuKα (λ = 1.54 Å) radiation source and an NaI scintillation counter detector. The X-ray source and detector for the pXRD defined a plane with the sample holder, and the slide surface was oriented perpendicular to said plane. The range between 2 and 50 2θ degrees was scanned, with a step size of 0.01° per 2 seconds. Samples included commercially obtained graphite, finely ground M_3HHTP_2, and M_3HHTP_2/graphite blends prepared according to the procedure detailed in Section 2.1. Homogenized powder samples were analyzed on a low background Silicon plate (MTI Corporation, Richmond, CA) on polymethylmethacrylate (PMMA) sample holders (~5 mg sample size). Random orientation of crystallites within the sample is assumed.

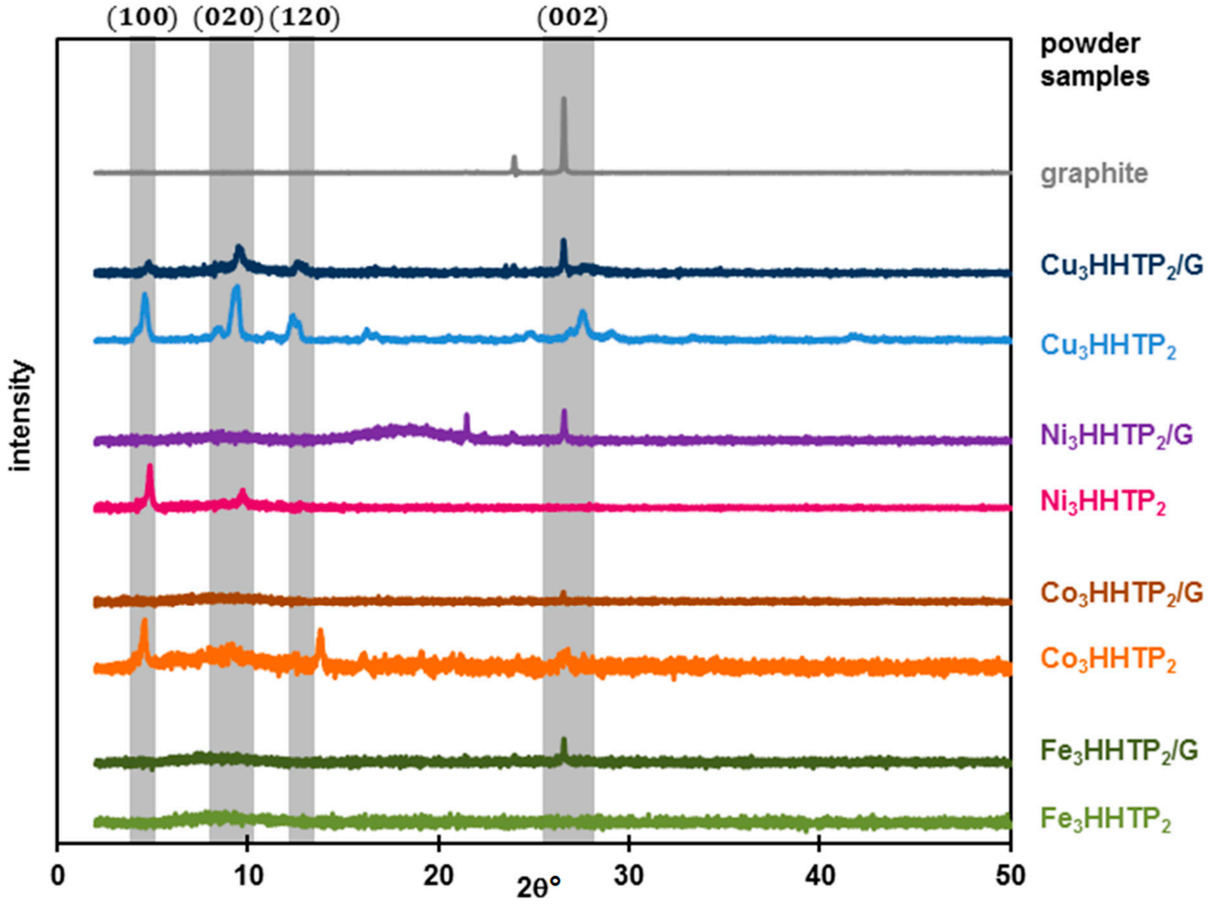

Figure S8. Powder X-Ray diffraction. Scaled powder X-Ray Diffraction (pXRD) spectra for graphite, M_3HHTP_2, and M_3HHTP_2/graphite blend bulk. The graphite peak at ~26° represents the interplanar (002) shear plane, corresponding to the stacking of 2D graphitic sheets. This peak is retained in each M_3HHTP_2/graphite blend, implying that graphite interplanar layers are not fully exfoliated upon ball-milling. Long-range crystallinity is diminished for the blends with the exception of Cu_3HHTP_2/G, which retains crystallinity after ball-milling with graphite. For Ni_3HHTP_2/G and Co_3HHTP_2/G, shear planes (100), (020), and (120) — all corresponding to Bragg planes perpendicular to the interplanar MOF layers — are attenuated upon ball-milling, suggesting significant loss of crystallinity upon milling. Fe_3HHTP_2 is amorphous in character before milling.

VIII. Thermal Gravimetric Analysis of MOFs

Thermal gravimetric analysis was performed using a TA Instruments TGA Q150 with a 10° C/min ramp from room temperature to 900° C.

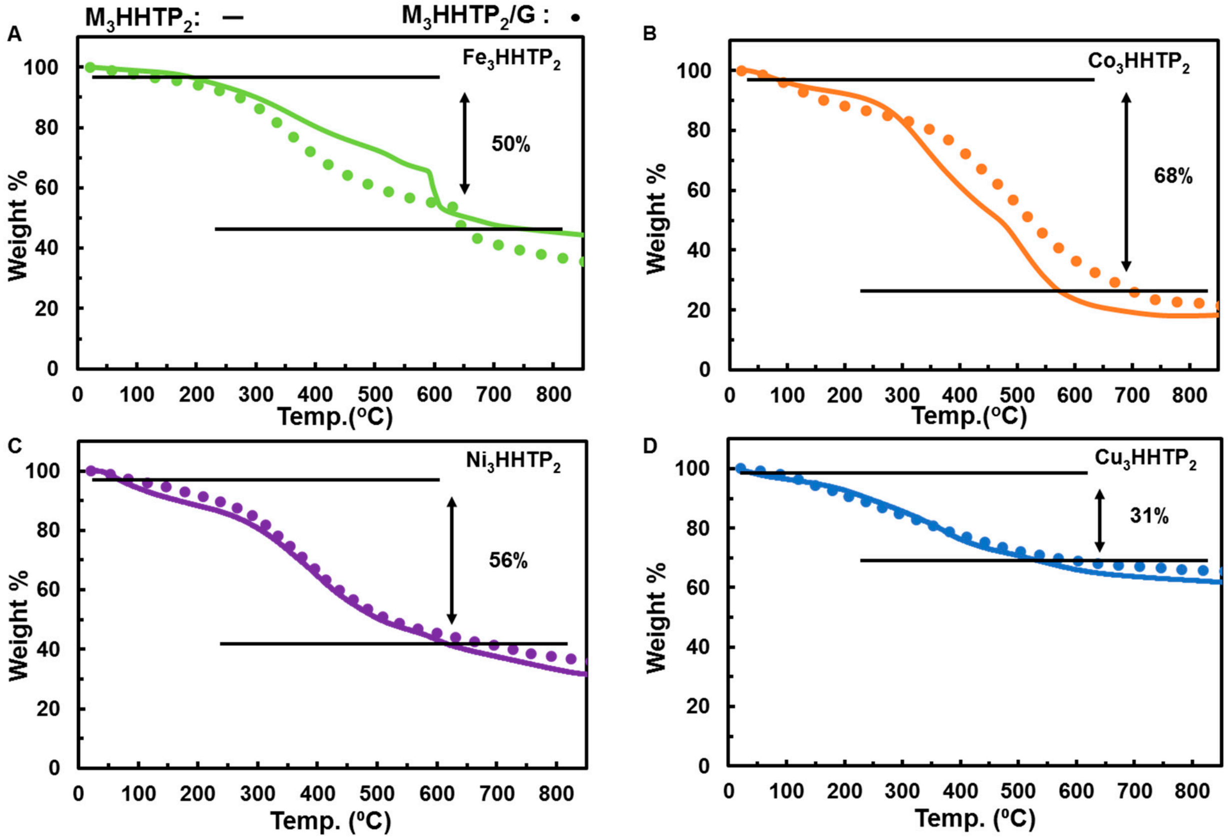

Figure S9. Thermal gravimetric analysis (TGA). TGA curves for M_3HHTP_2 are represented by a solid line and M_3HHTP_2/graphite blends are represented by a dotted line. A) Fe_3HHTP_2, B) Co_3HHTP_2, C) Ni_3HHTP_2, and D) Cu_3HHTP_2. A 2–3 % mass loss is observed at 100° C.

IX. Nitrogen Adsorption Measurements

A. Nitrogen Isotherms

We collected the adsorption measurements for Ni_3HHTP_2 and Cu_3HHTP_2 data using an ASAP Plus 2020 (Mircromeritics, Norcross, Georgia) with N_2 gas at 77K and the Co_3HHTP_2in and Fe_3HHTP_2 data using a 3flex™ Surface and Catalyst Characterization analyzer (purchased from Micromeritics). Samples were degassed under vacuum at 150° C from 180 minutes to 48 hours. For BET calculations, a fitting range of 0 to 0.3 P/P_o was used.

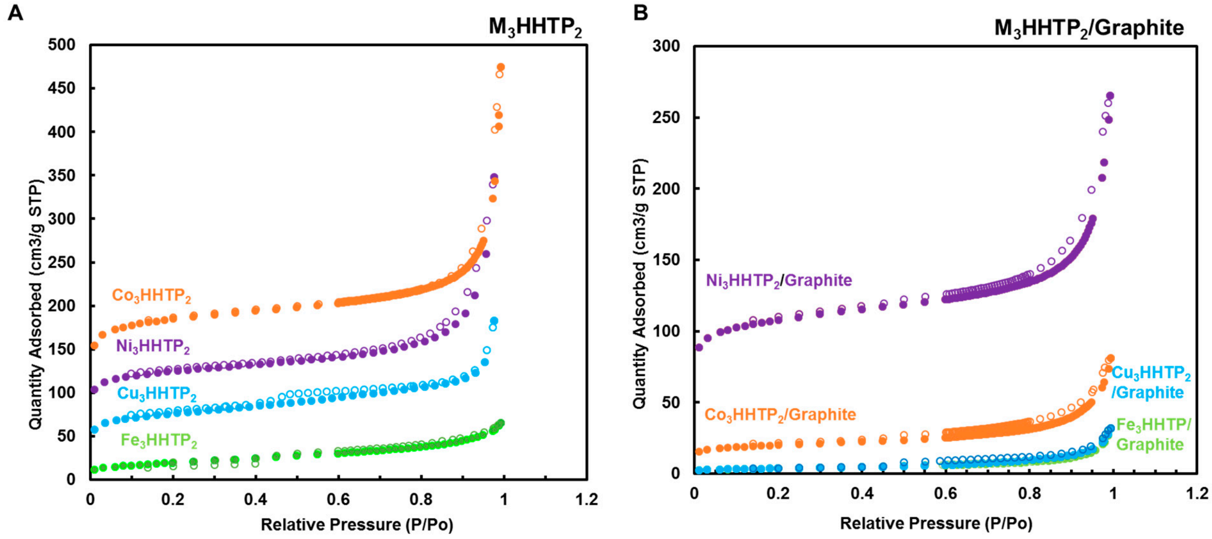

Figure S10. N_2 isotherm. A) The isotherm plot for Ni_3HHTP_2 is shown in purple, Cu_3HHTP_2 in blue, Co_3HHTP_2in orange and Fe_3HHTP_2 in green. The solid circle represents the adsorption plot whereas the open circle corresponds to the desorption plot. The significant uptake under 0.1 (P/P_o) is characteristic of a microporous material. The Brunauer-Emmet-Teller (BET) surface area for Ni_3HHTP_2 was calculated to be 473 m^2/g. BET surface area for Cu_3HHTP_2 was calculated to be 284 m^2/g. BET surface area for Co_3HHTP_2 was calculated to be 570 m^2/g. BET surface area for Fe_3HHTP_2 was calculated to be 69 m^2/g. The fitting range for BET calculations were 0 to 0.3 P/P_o. B**)** The BET adsorption analysis for Ni_3HHTP_2/graphite (purple), Cu_3HHTP_2/graphite (blue), Co_3HHTP_2/graphite (orange) and Fe_3HHTP_2/graphite (green). The BET surface area for each of the blends was 337 m^2/g, 13 m^2/g, 65 m^2/g, and 13 m^2/g, respectively.

Table S2. Table of BET surface areas for M_3HHTP_2 and M_3HHTP_2/Graphite

BET Surface Area (N_2) M_3HHTP_2 M=	Pure MOF M_3HHTP_2	Blended MOF M_3HHTP_2/Graphite
Cu	284 m^2/g	13 m^2/g
Ni	473 m^2/g	337 m^2/g
Co	571 m^2/g	65 m^2/g
Fe	69 m^2/g	13 m^2/g

B. T-Plot

Figure S11. A) The t-plot analysis (not fitted) using Harkins and Jura thickness equation for Ni_3HHTP_2 (purple), Cu_3HHTP_2 (blue), Co_3HHTP_2 (orange) and Fe_3HHTP_2 (green). B) The fitted t-plot analysis using the same thickness equation to calculate the external surface area. The external surface area for Ni_3HHTP_2 was calculated to be 79 m^2/g, 55 m^2/g for Cu_3HHTP_2, 158 m^2/g for Co_3HHTP_2 and 65 m^2/g for Fe_3HHTP_2. The blends exhibited decreased external surface areas of 118 m^2/g for Ni_3HHTP_2, 8.1 m^2/g for Cu_3HHTP_2, 33 m^2/g for Co_3HHTP_2 and 11 m^2/g for Fe_3HHTP_2.

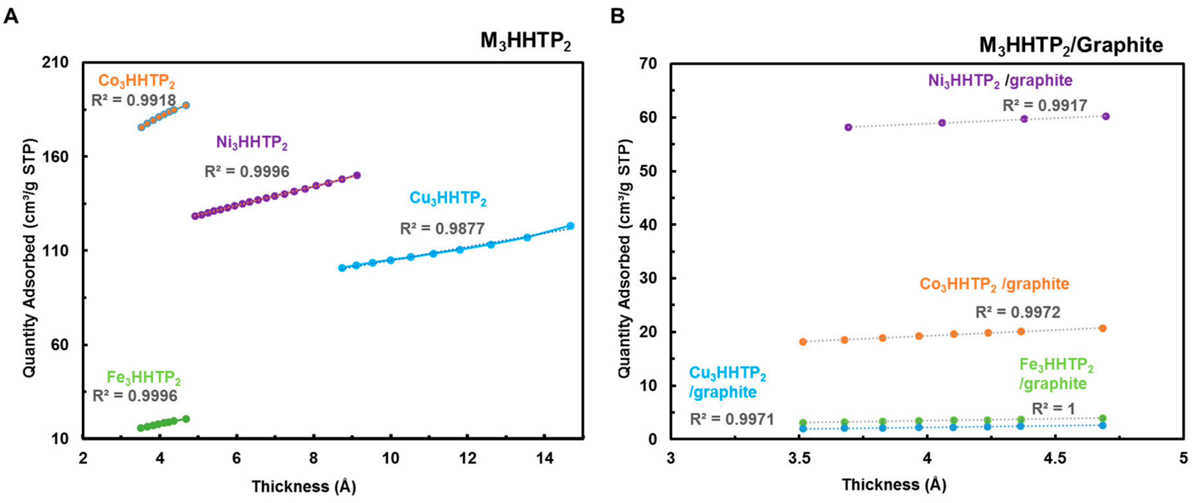

Table S3. Table of external surface areas for M_3HHTP_2 and M_3HHTP_2/Graphite

BET Surface Area (N_2) M_3HHTP_2 M=	Pure MOF M_3HHTP_2	Blended MOF M_3HHTP_2/Graphite
Cu	55 m^2/g	8.1 m^2/g
Ni	79 m^2/g	118 m^2/g
Co	158 m^2/g	33 m^2/g
Fe	65 m^2/g	11 m^2/g

X. Estimation of Thickness of the Abrasion Layer

We used equation **(2)** as a method for estimating thickness of the abrasion layers (t) for each drawn device on paper substrate. In this equation (m) signifies mass of each device, (ρ) represents density of the sensing material and A (cm^3) is the surface area of the sensing material after drawing.

$$t = [m/(\rho \times A)] \qquad \textbf{(2)}$$

We calculated thickness using density of pure graphite, pure MOF, and the weighted average of MOF and graphite blend ($0.9(\varrho_{MOF}) + 0.1(\varrho_{graphite})$). The density of graphite (Graphite powder, natural, microcrystal grade, APS 2-15 micron, 99.9995% (metals basis)) was calculated to be 2.224 g/cm^3, using the international union of crystallography site (http://checkcif.iucr.org/index.html) and the crystal structure file of a previously reported for graphite. The density of the MOF, equal to 1.589 g/cm^3, was calculated using the international union of crystallography site (http://checkcif.iucr.org/index.html) and the crystal structure file of a previously reported for Co_3HHTP_2 MOF.[1]

We used a microanalytical balance (with accuracy up to 1 μg) to measure the mass of a single paper chip containing four sensors before and after deposition of M_3HHTP_2/graphite blend by mechanical abrasion. To calculate *m*, we divided the mass of the blend on the surface of the paper chip by the number of sensors on the chip (n = 4).

We estimated *A* using the method described below.

1. An optical microscope (AmScope with Toupview software) was used to take high resolution (SNAP resolution - 2592 x 1944 with 10x magnification) images of the paper devices with each device previously drawn on.
2. ImageJ (Image processing and Analysis in Java) was used to estimate the area of M_3HHTP_2/graphite blend that covers one electrode (one device).
3. The colors of the images are split so that red pixels are removed to enhance contrast. We assumed the blue pixels corresponded sensing material, thus we calculated the total area of blue pixels (mm^2).

Table S4. Film Thickness of Materials on Devices. Table of areas, mass and density used in calculation for thickness of abraded layers.

**Paper device**	**Total area (cm^2)**	**A blue pixels (cm^2)**	**mass (μg)**	**ρ Graphite (g/cm^3)**	**ρ MOF (g/cm^3)**	**ρ Weighted average (g/cm^3)**	***t* Weighted average (μm)**	***t* MOF (μm)**	***t* Graphite (μm)**
**1**	3.7	2.3	27.5	2.22	1.6	1.7	0.39	0.40	0.29
**2**	3.5	2.3	27.5	2.22	1.6	1.7	0.38	0.40	0.28
**3**	4.1	0.5	27.5	2.22	1.6	1.7	0.53	0.56	0.40
**4**	2.7	0.1	27.5	2.22	1.6	1.7	0.20	0.22	0.15

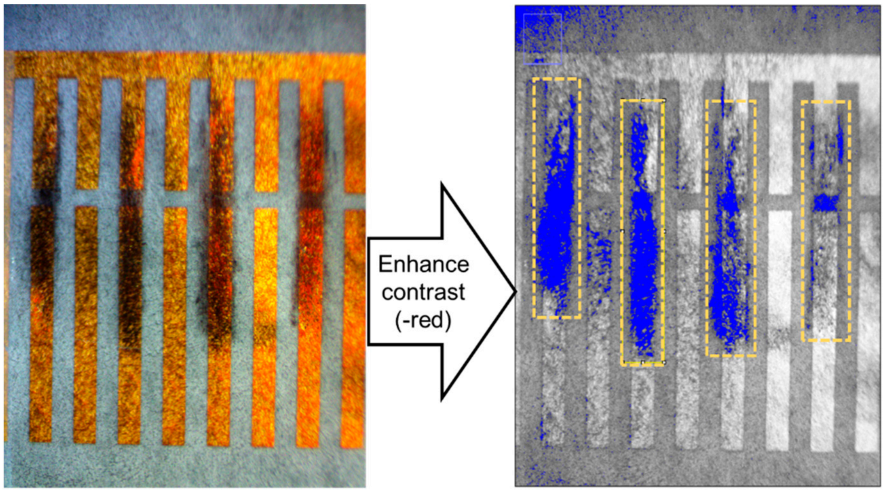

Figure S12. Enchantment of optical images to improve contrast of device image. Image taken with optical microscope and processed using ImageJ analysis to enhance contrast and calculate percentage of blue pixels for thickness.

XI. Current/Voltage Plots

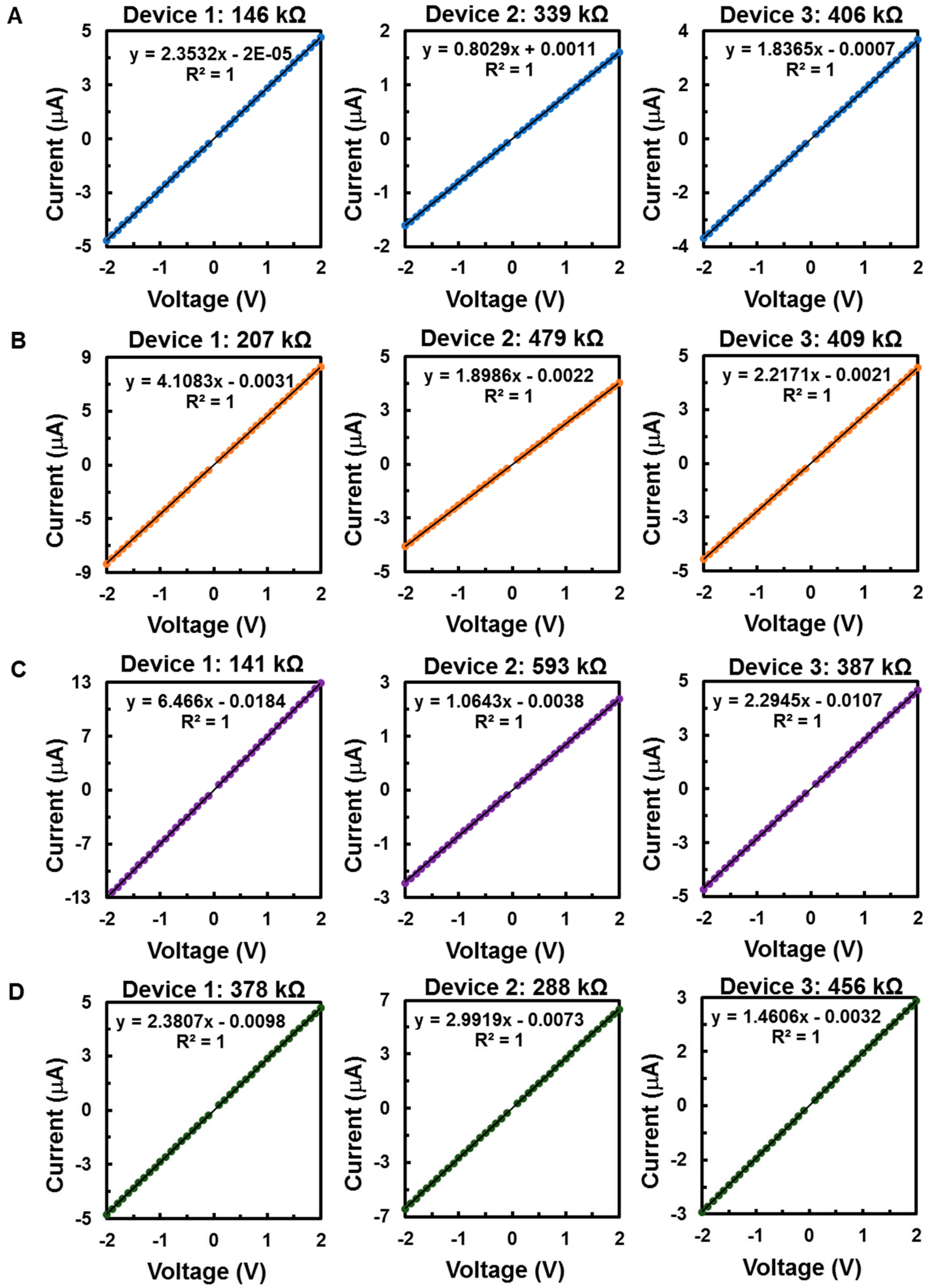

Figure S13. Current/voltage plots. Current/voltage plots demonstrate the ohmic behavior of the devices in the range of -2.0 V to 2.0 V. A) Cu_3HHTP_2/graphite blend device. B) Co_3HHTP_2/graphite blend device. C) Ni_3HHTP_2/graphite blend device. D) Fe_3HHTP_2/graphite blend device.

XII. Comparison in Sensing Performance of Pure MOF with Ball Milled MOF/Graphite Blends

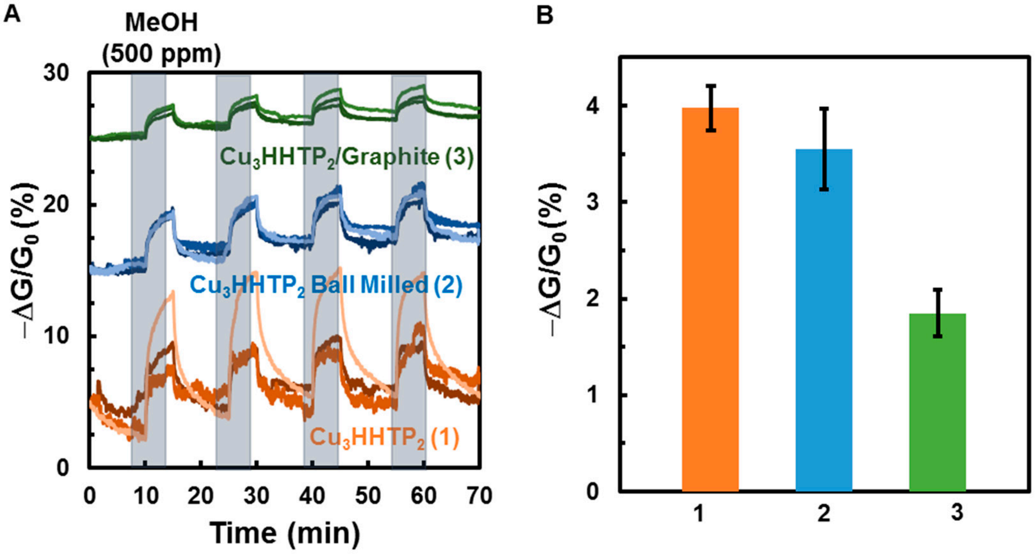

Figure S14. Plot comparing sensing performance of pure MOFs with ball milled blends integrated by abrasion into ceramic devices equipped with gold-interdigitated electrodes. A) Sensing trace representing the change in conductance $-\Delta G/G_0$ (%) over time (min) with pure Cu_3HHTP_2, ball-milled Cu_3HHTP_2, and Cu_3HHTP_2/graphite blend exposed to MeOH (500 ppm) diluted with N_2, using ceramic devices. B) Average sensing response of the three variants of copper MOF. Each bar represents the average value of response based on 4 exposures of 3 separate devices; the error bars represent the standard deviation from the average based on 4 exposures of 3 separate devices.

XIII. Analysis of Concentration Dependence

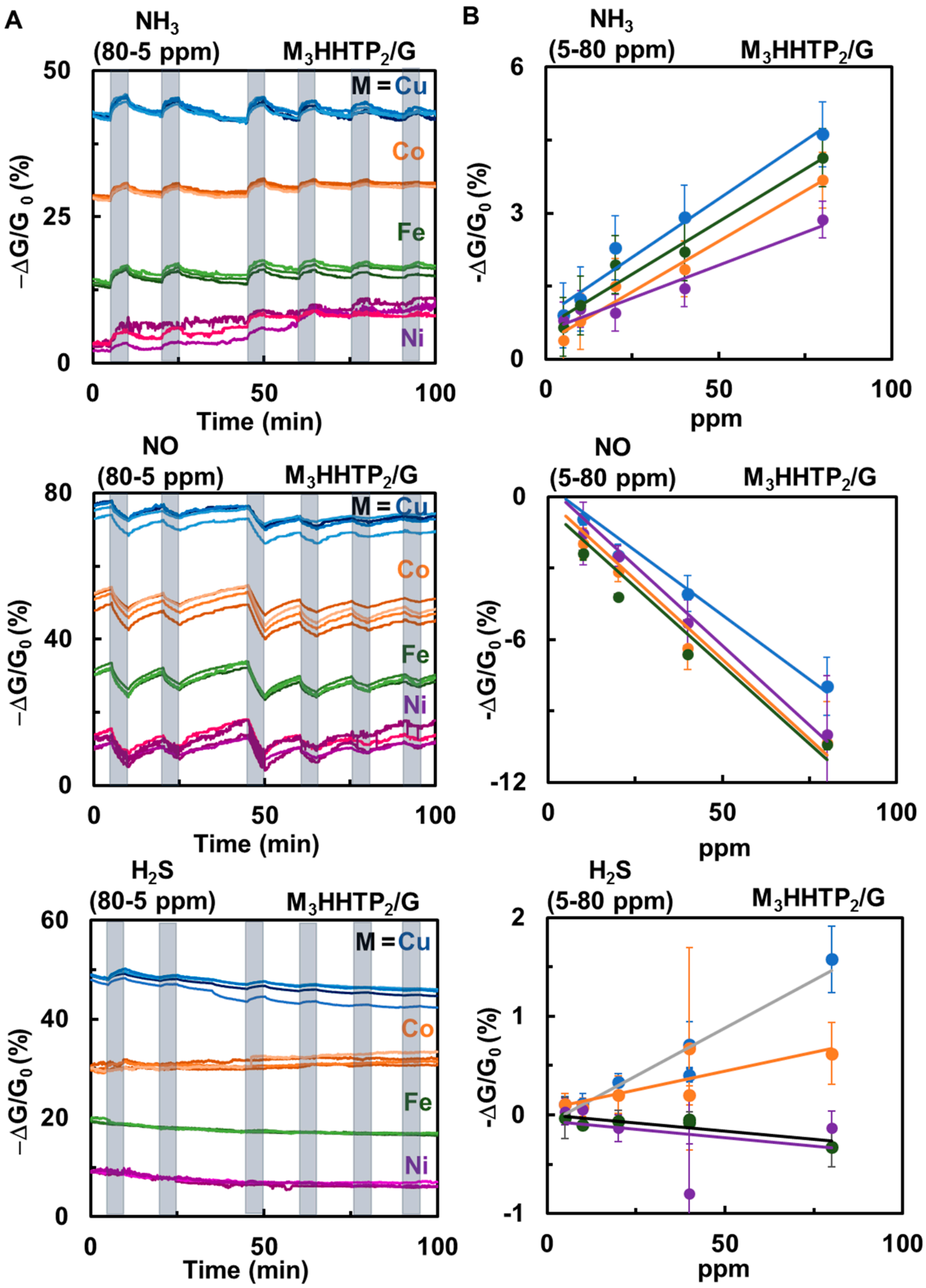

Figure S15. Continuous concentration dependence analysis on M_3HHTP_2/graphite blends with varying concentration of NH_3, NO, and H_2S (80-5ppm). A) Sensing performance of M_3HHTP_2/graphite blend array towards varying concentrations of NH_3, NO, and H_2S (80, 40, 20, 10, 5 ppm) diluted with N_2, exposed for five-minutes and 10-minute recovery times. A longer baseline is seen between the second and third exposures to allow for a proper baseline formation during a change from 0.5 L/min to 1.0 L/min. B) Plot of sensing response of the M_3HHTP_2/ graphite blends with respect to NH_3, NO, and H_2S at 5-80ppm. Each dot represents the average value of response based on 4 exposures of 3 separate devices; the error bars represent the standard deviation from the average.

XIV. Saturation Response of Sensor Array with NH_3

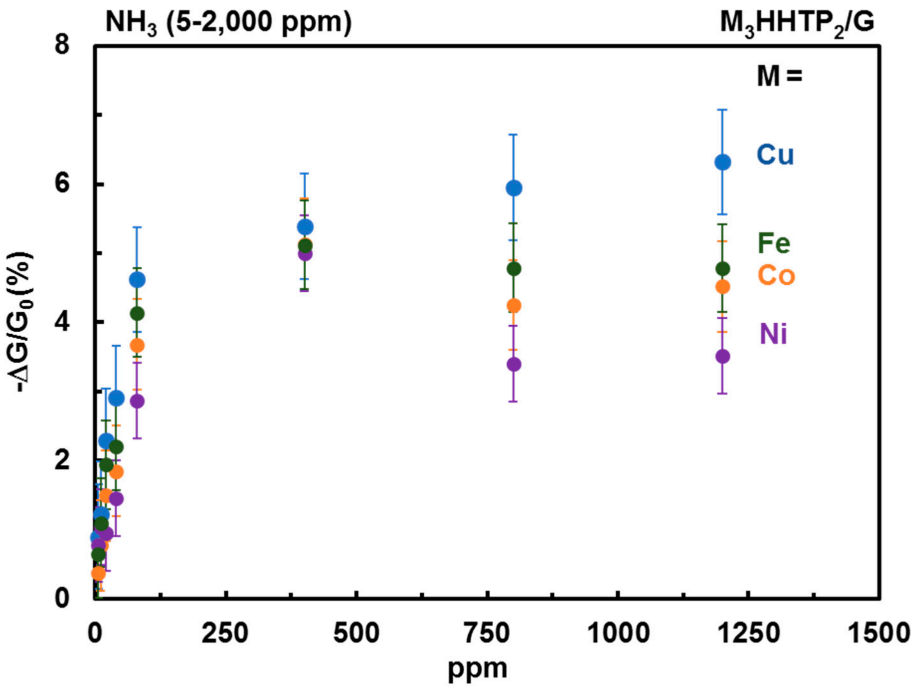

Figure S16. Saturation analysis on M_3HHTP_2/graphite blends with varying concentration of NH_3 (5-8,000 ppm). Sensing performance of M_3HHTP_2/graphite blend array towards varying concentrations of NH_3 (2,000, 1,600, 1,200, 800, 80, 40, 20, 10, 5 ppm) diluted with N_2, exposed for five-minutes and 10-minute recovery times. A linear increase in response is observed through 80 ppm NH_3 exposure with a saturation limit occurring after 80 ppm NH_3 exposure. Subsequent exposures after 80 ppm NH_3 only show a very small increase in response with higher doses of NH_3.

XV. Response of Sensor Arrays Comprising of MOF/Graphite Blends to Additional Gases and Vapors

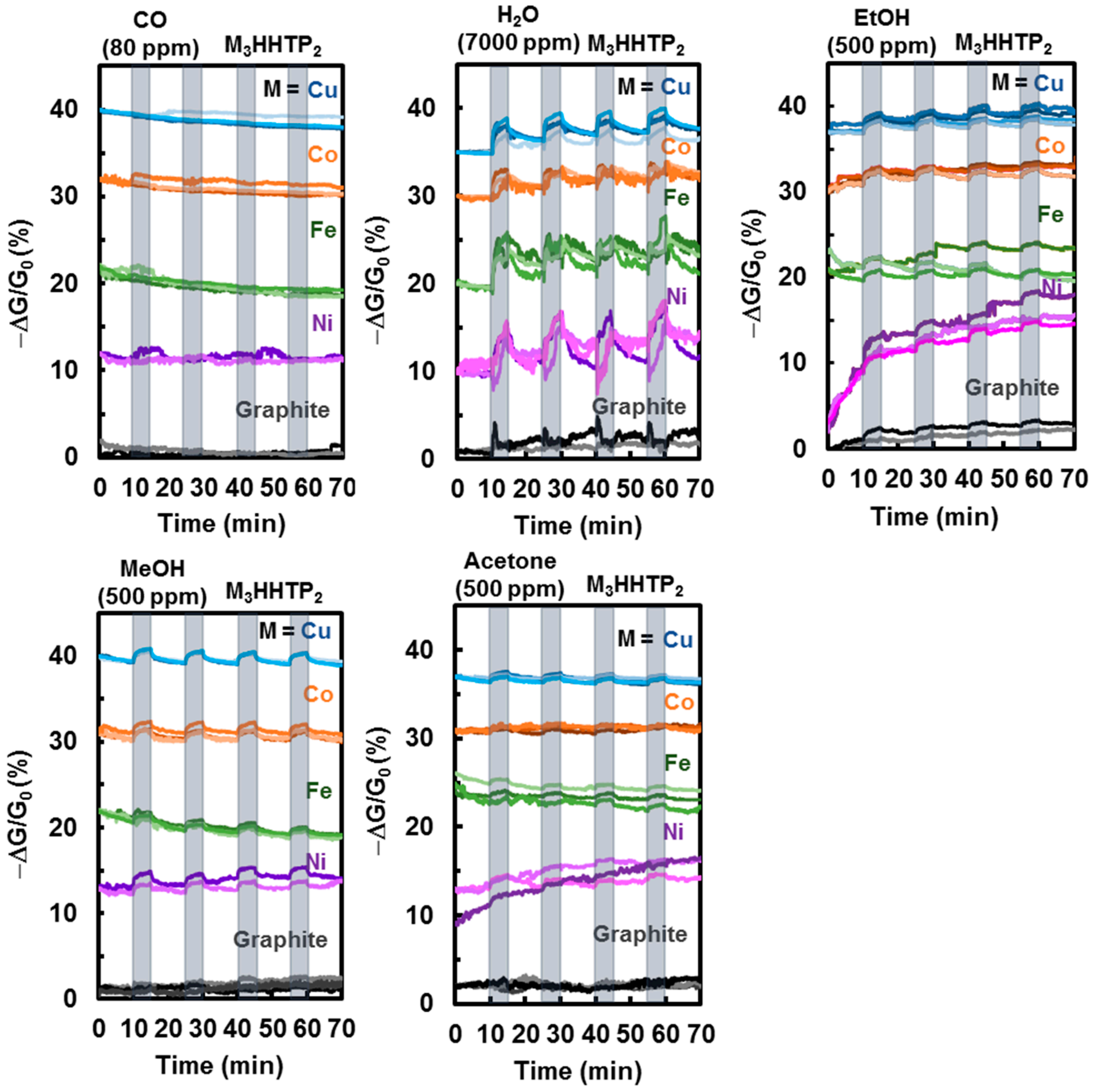

Figure S17. Plots showing response of arrays to different gases and vapors. Sensing performance of chemiresistive device array towards gaseous analytes. Sensing trace representing the change in conductance -Δ G/G0 (%) over time (min) with the M_3HHTP_2/graphite blends exposed to CO (80 ppm), EtOH, MeOH, and acetone (500 ppm), and H_2O (7000 ppm) diluted with N_2.

XVI. Batch-to-Batch Influence of MOF/Graphite Blend for Chemiresistive Sensing

Batch 1 and Batch 2 of Cu_3HHTP_2 was synthesized using a 200 mg scale (HHTP). Both batches were blended with graphite to form the blend. Three devices of batch 1 were fabricated by mechanical abrasion onto paper devices with gold electrodes and exposed to MeOH (500 ppm) followed by NH_3 (80 ppm) with four five-minute exposures and four 10-minute recovery periods. Similarly, three more devices of batch 2 were fabricated by mechanical abrasion onto paper devices with gold electrodes and the same test was performed.

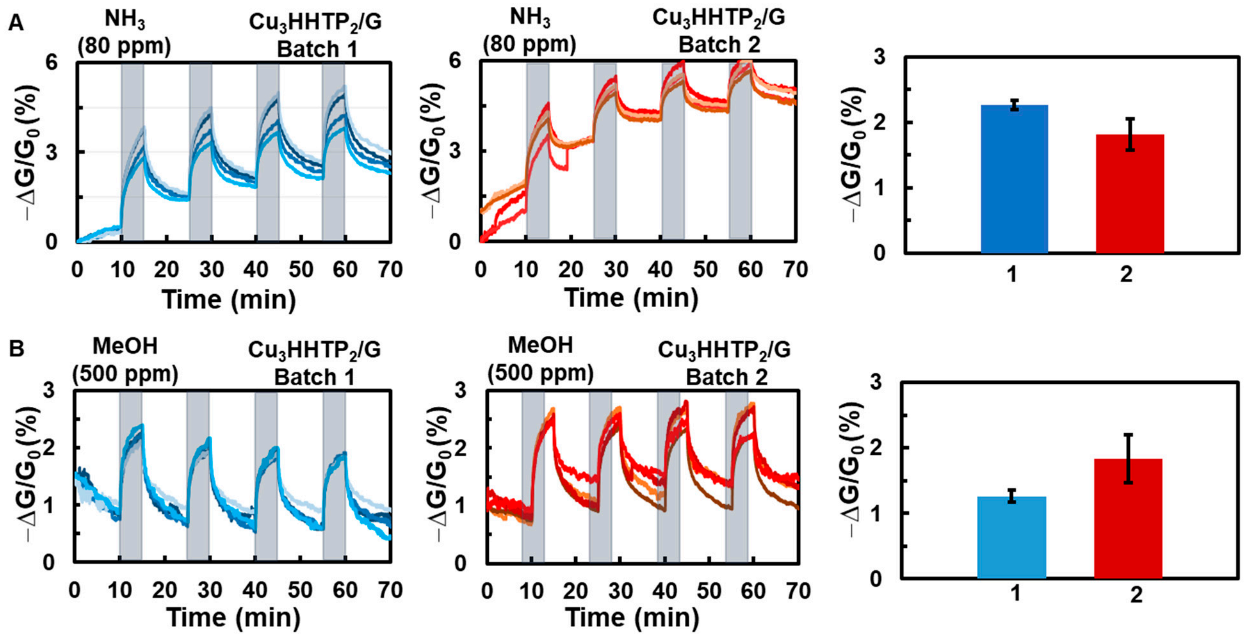

Figure S18. Plot showing batch-to-batch reproducibility of chemiresistive sensors of M_3HHTP_2/graphite blends abraded between gold electrodes on paper. Sensing trace representing the change in conductance -Δ G/G_0 (%) over time (min) with Cu_3HHTP_2/graphite blend abraded between gold electrodes on paper devices followed by subsequent exposure to NH_3 (80 ppm) and MeOH (500 ppm). Blue represents the first batch (4 exposures with 3 devices) and red represents second batch (4 exposures with 3 devices). Average sensing response of Cu_3HHTP_2/graphite blend is plotted onto a bar graph with each bar representing the average percent response changed based on 4 exposures of 3 devices. The error bars represent the standard deviation from the average. A) Exposure to NH_3 (80 ppm). B) Exposure to MeOH (500 ppm).

XVII. Scale-Dependent Cu_3HHTP_2 MOF Morphology and Sensing Response

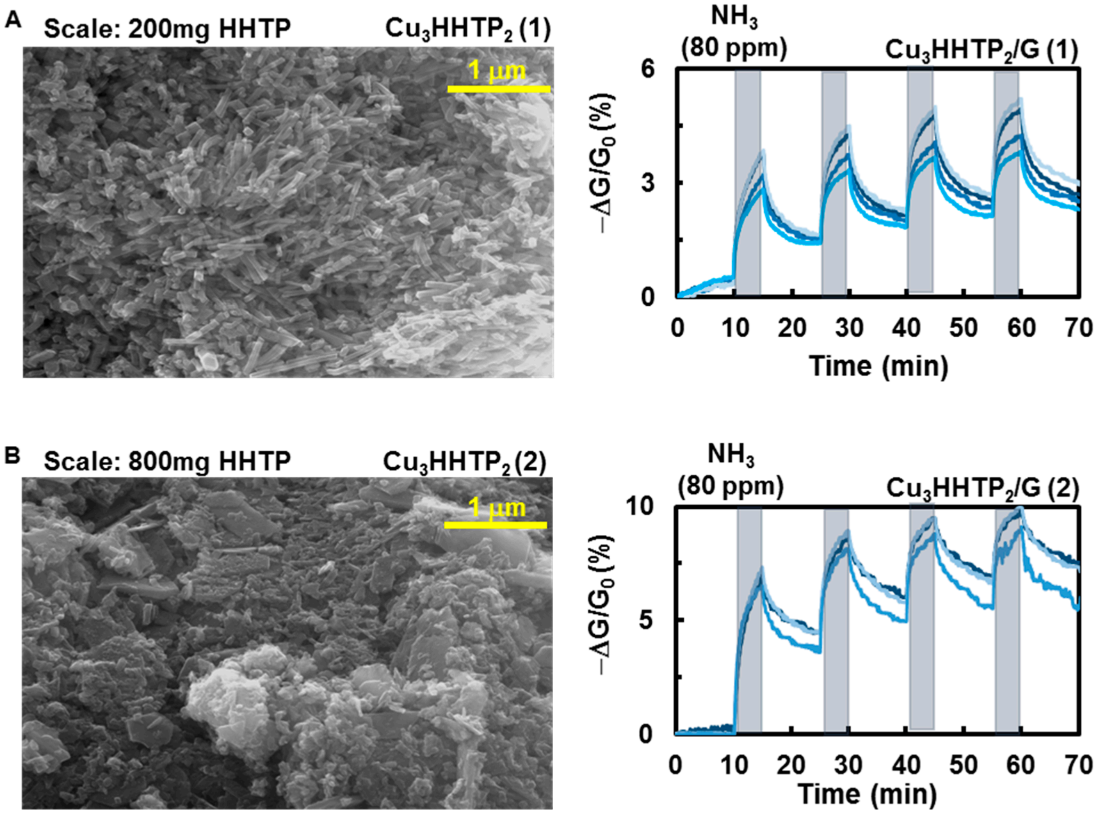

Figure S19. Scale dependence of Cu_3HHTP_2 MOF morphology and sensing response. A) Small scale (200 mg of HHTP) Cu_3HHTP_2 MOF reaction shows an SEM image with nanorod morphology. Sensing trace shows a decrease in conductance with an average of 2.5% ± 0.2% change. B) Large scale (800 mg of HHTP) Cu_3HHTP_2 MOF reaction shows an SEM image with flake and small chunk morphology. Sensing trace shows a decrease in conductance with an average of 3.7% ± 0.6% change.

XVIII. Influence of Previous Analyte Exposure on Subsequent Sensing Performance

Cu_3HHTP_2 was synthesized and ball-milled with graphite to form the blend. For preconditioned devices, three devices were fabricated and exposed to MeOH (500 ppm) with five-minute exposures and 10-minute recovery periods. Immediately after, the devices were exposed to NH_3 with five-minute exposures and 10-minute recovery periods. No preconditioning exposed the devices immediately to NH_3 (80 ppm).

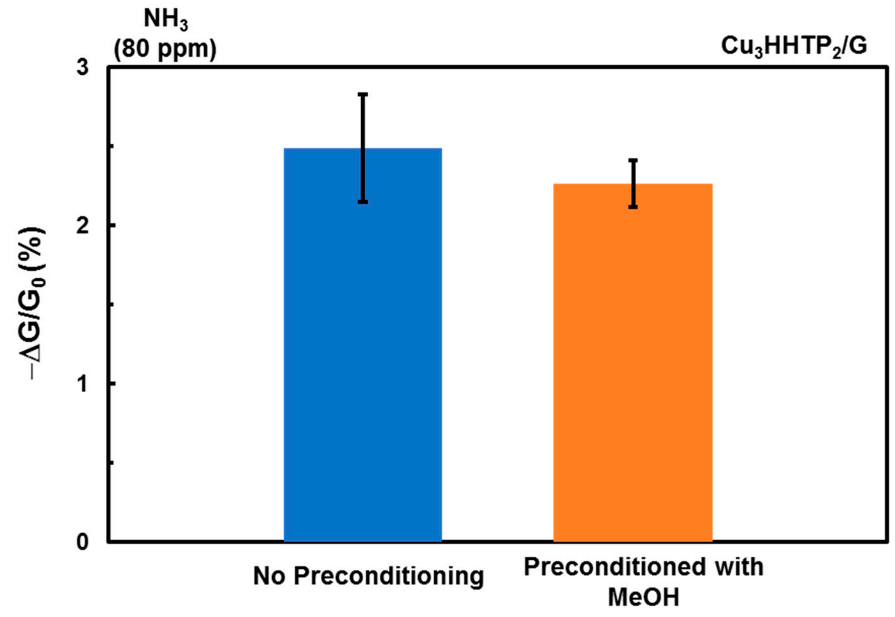

Figure S20. Average response plot of Cu_3HHTP_2 MOF with no preconditioning versus preconditioning. Cu_3HHTP_2/graphite blend devices exposed to NH_3 before or after MeOH exposure has no substantial difference. No precondition has blend devices exposed to NH_3 first. Preconditioned blend devices are exposed to MeOH in a standard exposure trial (four exposures of 10 minutes with three recovery periods of five-minutes).

XIX. Principle Component Analysis

Table S5. Average sensory response for three arrays, excluding first exposures and graphite.

Array	M_3HHTP_2 /G M=	80 ppm NH_3	80 ppm NO	80 ppm H_2S	7000 ppm H_2O
Array #1 (-ΔG/G_0)	Cu	2.41	-1.67	0.95	2.12
	Ni	1.31	-1.77	0.44	2.38
	Co	2.78	-2.45	0.01	1.27
	Fe	2.40	-1.66	-0.23	1.41
Array #2 (-ΔG/G_0)	Cu	2.34	-1.55	0.68	0.97
	Ni	1.10	-1.96	0.01	2.20
	Co	2.67	-2.42	0.25	0.96
	Fe	2.27	-1.65	0.13	2.29
Array #3 (-ΔG/G_0)	Cu	2.12	-0.84	0.65	1.86
	Ni	1.17	-1.00	0.24	4.10
	Co	2.55	-1.36	0.23	0.56
	Fe	2.12	-0.90	0.35	1.83

Table S6. Principle Component scores for the three arrays featured in Table S4, high concentration of analyte.

Principle Component Scores			
	**Analyte**	**PC1 (95%)**	**PC2 (5%)**
Array #1	80 ppm NH_3	3.25	1.14
	80 ppm NO	-4.99	-0.03
	80 ppm H_2S	-0.63	-0.36
	7000 ppm H_2O	2.36	-0.74
	**Analyte**	**PC1 (93%)**	**PC2 (7%)**
Array #2	80 ppm NH_3	3.17	0.94
	80 ppm NO	-4.83	-0.07
	80 ppm H_2S	-0.50	0.39
	7000 ppm H_2O	2.17	-1.26
	**Analyte**	**PC1 (85%)**	**PC2 (15%)**
Array #3	80 ppm NH_3	2.32	-1.33
	80 ppm NO	-3.72	-0.38
	80 ppm H_2S	-0.94	-0.50
	7000 ppm H_2O	2.34	2.21

XX. Variance Device:Device and Batch:Batch

Table S7. Average sensory response for Cu_3HHTP_2/Graphite, excluding first exposures and graphite.

Cu_3HHTP_2/graphite average exposure 3 x 80 ppm ($-\Delta G/G_0$)						
**Device**	**Batch 1**			**Batch 2**		
	**NH_3**	**NO**	**H_2S**	**NH_3**	**NO**	**H_2S**
**1**	4.62	-7.97	0.95	2.41	-1.67	1.69
**2**	3.96	-6.07	0.68	2.31	-1.55	0.86
**3**	3.38	-4.80	0.65	2.12	-0.84	1.08
**Avg**	3.99	-6.28	0.76	2.29	-1.36	1.21
**St.dev**	0.62	1.59	0.16	0.15	0.45	0.43
**analyte specific variance**	15.5%	25.4%	21.6%	**6.65%**	**33.1%**	**35.5%**
**batch variance**	**overall coefficient of variance for batch 1 = 20.1%**					
				**overall coefficient of variance for batch 2 = 25.1%**		
**overall coefficient of variance batch:batch = 42.1%**						

Table S8. Average sensory response for Ni_3HHTP_2/Graphite, excluding first exposures and graphite.

Ni_3HHTP_2/graphite average exposure 3 x 80 ppm ($-\Delta G/G_0$)						
**Device**	**Batch 1**			**Batch 2**		
	**NH_3**	**NO**	**H_2S**	**NH_3**	**NO**	**H_2S**
**1**	2.86	-10.00	not applicable (no response)	1.31	-1.77	not applicable (no response)
**2**	1.77	-6.38		1.10	-1.96	
**3**	2.51	-6.05		1.17	-1.00	
**Avg**	3.99	-6.28		2.29	-1.36	
**St.dev**	0.62	1.59		0.15	0.45	
**analyte specific variance**	23.4%	29.3%		9.02%	33.3%	
**batch variance**	**overall coefficient of variance for batch 1 = 26.4%**					
				**overall coefficient of variance for batch 2 = 20.7%**		
**overall coefficient of variance batch:batch = 44.5%**						

Table S9. Average sensory response for Co_3HHTP_2/Graphite, excluding first exposures and graphite.

Co_3HHTP_2/graphite average exposure 3 x 80 ppm ($-\Delta G/G_0$)						
**Devices**	**Batch 1**			**Batch 2**		
	**NH_3**	**NO**	**H_2S**	**NH_3**	**NO**	**H_2S**
**1**	3.68	-10.33	not applicable (no response)	2.78	-2.45	not applicable (no response)
**2**	2.82	-7.03		2.67	-2.42	
**3**	2.52	-6.13		2.55	-1.36	
**Avg**	3.01	-7.83		2.66	-2.08	
**St.dev**	0.60	2.21		0.12	0.62	
**analyte specific variance**	20.0%	28.2%		4.33%	29.8%	
**batch variance**	**overall coefficient of variance for batch 1 = 24.1%**					
				**overall coefficient of variance for batch 2 = 17.1%**		
**overall coefficient of variance batch:batch = 35.0%**						

Table S10. Average sensory response for Fe_3HHTP_2/Graphite, excluding first exposures and graphite.

Fe_3HHTP_2/graphite average exposure 3 x 80 ppm ($-\Delta G/G_0$)						
**Device**	**Batch 1**			**Batch 2**		
	**NH_3**	**NO**	**H_2S**	**NH_3**	**NO**	**H_2S**
**1**	4.14	-10.42	not applicable (no response)	2.40	-1.66	not applicable (no response)
**2**	3.40	-7.91		2.27	-1.65	
**3**	2.60	-7.07		2.12	-0.90	
**Avg**	3.50	-8.47		2.26	-1.40	
**St.dev**	0.60	1.74		0.14	0.44	
**analyte specific variance**	17.0%	20.6%		6.21%	31.2%	
**batch variance**	**overall coefficient of variance for batch 1 = 18.8%**					
				**overall coefficient of variance for batch 2 = 18.7%**		
**overall coefficient of variance batch:batch = 38.7%**						

XXII. Signal-to-Noise Analysis on Chemiresistive Response of Cu_3HHTP_2 and Cu_3HHTP_2/Graphite

We calculated the signal-to-noise ratio (SNR) of Cu_3HHTP_2, Cu_3HHTP_2 ball-milledCu_3HHTP_2/graphite sensors (data from **Figure S14**) using the root-mean-square (rms) deviation in conductance from the baseline to exposure of the analytes. For each sensor (pure, ball-milled and blended) we took 20 consecutive points prior to exposure and fit a fifth order polynomial using Microsoft excel. We then used equation (3) to calculate $V_x{}^2$ and $rms_{noise.}$ To calculate SNR we divided the average magnitude of the reponse ($-\Delta G/G_o$) by rms_{noise} and obtained the values in the table below.

$$
\begin{aligned}
Vx^2 &= \sum (yi - y)\ x^2 \\
rms_{noise} &= \sqrt{(Vx^2)} / N \\
SNR &= (-\Delta G/G_o) / rms_{noise}
\end{aligned}
\tag{3}
$$

Table S11. Signal-to-noise ratios of Cu_3HHTP_2/graphite, Cu_3HHTP_2 Ball-milled, and Cu_3HHTP_2.

Device	Cu_3HHTP_2/Graphite	Cu_3HHTP_2 Ball-Milled	Cu_3HHTP_2
**1**	6.94	18.55	1.78
**2**	3.67	45.0	7.61
**3**	5.88	0.86	11.35
**Avg.**	5.5	21.5	8.95
**Std. Dev.**	1.36	18.12	5.68

XXIII. References

1. Hmadeh, M.; Lu, Z.; Liu, Z.; Gándara, F.; Furukawa, H.; Wan, S.; Augustyn, V.; Chang, R.; Liao, L.; Zhou, F.; Perre, E.; Ozolins, V.; Suenaga, K.; Duan, X.; Dunn, B.; Yamamto, Y.; Terasaki, O.; Yaghi, O. M., New Porous Crystals of Extended Metal-Catecholates. *Chem. Mater.* **2012**, *24*, 3511–3513.
